# Supplementary material for: A spatial human thymus cell atlas mapped to a continuous tissue axis
Source: Nature. 2024 Nov 20;635(8039):708–18. doi: 10.1038/s41586-024-07944-6 (PMC11578893; doi:10.1038/s41586-024-07944-6)
Supplement: Supplementary file 1 — Supplementary Notes: explanations on TissueTag-based image annotation, OrganAxis establishment and details on scRNA-seq annotation and mapping. Supplementary Discussion: additional context for the findings in this study. Supplementary Figs. 1–19: details are provided in the Supplementary Notes and Methods. [file 41586_2024_7944_MOESM1_ESM.pdf]

nature portfolio

https://doi.org/10.1038/s41586-024-07944-6

Supplementary information

A spatial human thymus cell atlas mapped to a continuous tissue axis

In the format provided by the authors and unedited

Supplementary Information for:

A spatial human thymus cell atlas mapped to a continuous tissue axis

Nadav Yayon^, Veronika R. Kedlian^, Lena Boehme^, Chenqu Suo, Brianna T. Wachter, Rebecca T. Beuschel, Oren Amsalem, Krzysztof Polanski, Simon Koplev, Elizabeth Tuck, Emma Dann, Jolien Van Hulle, Shani Perera, Tom Putteman, Alexander V. Predeus, Monika Dabrowska, Laura Richardson, Catherine Tudor, Alexandra Y. Kreins, Justin Engelbert, Emily Stephenson, Vitalii Kleshchevnikov, Fabrizio De Rita, David Crossland, Marita Bosticardo, Francesca Pala, Elena Prigmore, Nana-Jane Chipampe, Martin Prete, Lijiang Fei, Ken To, Roger A. Barker, Xiaoling He, Filip Van Nieuwerburgh, Omer Ali Bayraktar, Minal Patel, E. Graham Davies, Muzlifah A. Haniffa, Virginie Uhlmann, Luigi D. Notarangelo, Ronald N. Germain*, Andrea J. Radtke*, John C. Marioni*, Tom Taghon*, Sarah A. Teichmann*

Supplementary Notes **2**

Supplementary Note 1: Image annotation with TissueTag 2

Supplementary Note 2: Establishment of OrganAxis for CCF construction 3

The need for a CCF 4

Prerequisites for the establishment of a CCF 4

The OrganAxis model 5

Model derivation for the CMA 7

Supplementary Note 3: Immune and stromal cell types in the scRNA-seq dataset 9

Supplementary Note 4: Annotation of T lineage differentiation stages in fetal and paediatric scRNA-seq data 11

Supplementary Note 5: Localisation of paediatric mcTECs is associated with differential priming towards cTECI vs. mTECI fate 12

Supplementary Note 6: CITE-seq permits fine grained annotation and high-resolution spatial mapping of the T lineage 13

Supplementary Discussion **15**

Supplementary References **18**

Supplementary Figures **20**

Supplementary Notes

Supplementary Note 1: Image annotation with TissueTag

TissueTag is a Jupyter-based image annotation tool that utilises the Bokeh Python library (http://www.bokeh.pydata.org) for interactive annotation of various image types at a user-defined resolution (e.g., brightfield, fluorescence). Image annotation can be carried out (semi-)automatically to reduce human operator error and increase robust comparisons between different samples and spatial technologies. Several modes of annotation are offered depending on the type of data and tissue complexity (https://github.com/Teichlab/TissueTag).

Visium data is processed using Space Ranger software (10x Genomics), which imposes a 2000 x 2000 pixel limit that is insufficient for high-resolution annotation. We developed a Visium data preprocessing pipeline which enables the retrieval of the reference H&E image at the highest resolution available (we used 5000 x 5000 pixels or 0.5 pixels per µm). This is followed by *de novo* alignment to the fiducial frame by affine transformation and tissue region detection with an adjustable intensity threshold (**Supplementary Figure 1a**, see Methods).

To match Visium H&E, TissueTag can generate a virtual H&E image based on two user-defined fluorescent channels through an implementation adapted from Simonson and colleagues[63]. In this study we selected Pan-Cytokeratin and Hoechst nuclear stain for the generation of IBEX virtual H&E images to highlight the major structures for annotation (cortex, medulla, capsule, Hassall's corpuscles (HC), perivascular space (PVS)) (**Supplementary Figure 1b,** see Methods).

To minimise human error during image annotation across sections, a pixel random forest (RF) classifier is trained either using Visium spots where a marker gene is highly expressed as masks or manual masks by "scribbling" of tissue annotations on easy-to-call regions which is more globally applicable (for Visium an IBEX alike) (**Supplementary Figure 1c**). Pixel RF predictions can subsequently be corrected and additional fine structure annotations can be added manually (**Supplementary Figure 1d**). All annotation metadata, such as region names, annotation resolution, pixels per µm, as well as pixel value interpretation of annotation names (e.g., 1 = "Medulla") and colours (e.g., "Medulla": "magenta") can be saved as .pkl files for easy recovery and reproducibility across a dataset. Note that multiple annotation layers can be made for the same reference image as needed.

In the present study, all Visium samples were annotated with TissueTag using the semi-automatic mode to consistently call the border between cortex and medulla. Cortex and medulla pixels were predicted with a pixel RF classifier by generating training annotations

based on spots that showed the highest gene expression of *AIRE* (for medulla) and *ARPP21* (for cortex). Next, the manual annotation tool (“annotator”) from TissueTag was used to adjust automatic cortex/medulla annotations where necessary as well as draw annotations for the specific structures, such as “capsule”/”edge”, freezing/sectioning artefacts, HCs, PVS, and fetal thymus-associated lymphoid aggregates (TALA). Finally, individual lobules were marked using the “poly_annotator” function. For IBEX samples, training labels for cortex vs. medulla were generated manually via “scribbling” on the virtual H&E image and then predictions for the full pixel space were obtained with a pixel RF classifier. The following fine structure and lobule annotations were performed on IBEX virtual H&E images as described for Visium data.

Discrete tissue annotations produced with TissueTag are the basis for L2 distance measurements - the minimal Euclidean distance of a spot in space to a discrete structure using nearest KNN points (e.g., cortex or medulla) (**Supplementary Figure 1e**, Methods). These distance measurements are also used to calculate a morphological axis (OrganAxis, more details in **Supplementary Note 2**, Methods). All annotations can be mapped back to Visium spots or IBEX cells for downstream analysis.

Supplementary Note 2: Establishment of OrganAxis for CCF construction

This supplementary note provides detailed explanations on the use of TissueTag and the establishment of an OrganAxis. We have also generated a complementary online tutorial and recommend following this resource for most up to date implementations and illustrations: https://organ-axis-tutorial.readthedocs.io/en/latest/index.html#

OrganAxis is a computational approach for the construction of a continuous axis that can serve as a Common Coordinate Framework (CCF) for an organ/tissue from a set of tissue landmarks.

Key features of OrganAxis:

- Derived purely from the reference image. This makes it universally applicable across virtually any spatial platform and at any resolution.
- Straightforward approach that significantly reduces the risk of double-dipping (e.g. it is not derived from high-dimensional gene space).
- Allows multisample “diagonal” integration while preserving continuous spatial information.

- Allows modelling of linear and non-linear spatial associations to account for local and global tissue environments, e.g. cellular neighbourhoods and anatomical structures.
- Hypothesis-driven: Prior knowledge about the tissue in question is needed to derive a biologically robust and meaningful axis.

OrganAxis is implemented to derive the human thymus cortico-medullary axis (CMA) here: https://github.com/Teichlab/thymus_spatial_atlas/tree/main

The need for a CCF

A Common Coordinate Framework (CCF) is a tool used by researchers and physicians to map specimens into a unified reference space. CCFs come in various types and complexities depending on the level of variability between specimens[14]. For example, some CCFs rely on morphological, histological, or molecular landmarks for orientation, while others utilise non-linear transformations to align different specimens. Some systems even combine both landmark-based methods and non-linear warps. A central example of an organ-specific CCF is the Allen Mouse Brain Atlas (https://mouse.brain-map.org/static/atlas) which is particularly effective due to the relatively low variability between mouse brain samples, allowing 2D tissue sections to be mapped to the 3D brain space of a typical mouse. In contrast, the human brain presents significant challenges due to the considerable variability between individuals. To address this, advanced mapping algorithms that employ non-linear and statistical 3D transformations are necessary[64]. The concept of using the vasculature system as a CCF[65] for the Human Reference Atlas has been proposed by the Human BioMolecular Atlas Program[66], and the Gut Linear Model has been developed for the human gut cell atlas[67]. However, there is a distinct lack of CCFs for the majority of human organs, which prevents knowledge synthesis from multiple sources and cross-consortia efforts.

Prerequisites for the establishment of a CCF

Consistent tissue annotations

We identified a significant gap in the ability to obtain robust tissue annotations across various spatial technologies. For instance, Visium spatial transcriptomics data (10x Genomics) is commonly annotated using the 10x Genomics Loupe Browser (https://www.10xgenomics.com/support/software/loupe-browser/latest), which limits annotations to individual Visium spots with a diameter of 55 μm and a 100 μm centre-to-centre distance between the spots. For high-resolution imaging technologies, such as IBEX[53], users may employ tools like Napari (https://napari.org/stable/), where annotations can be made at the pixel level and specific plugins for different spatial technologies are

being developed continuously. Nonetheless, the use of external software presents challenges for tissue annotations on high-performance computing infrastructure, which is regularly used by bioinformaticians. These challenges motivated us to create TissueTag for interactive tissue annotation within a Jupyter notebook environment (https://github.com/Teichlab/TissueTag).

Spatial sampling resolution

To develop a CCF that facilitates comparison across multiple modalities, it is crucial to precisely define the spatial sampling frequency/resolution of the tissue and consistently maintain it across all samples and modalities. Spatial sampling frequency (*r*) is defined in µm or mm (e.g., spot every 15 µm) and is independent of the imaging resolution, which is defined in pixel space (**Supplementary Figure 2a**). To harmonise sampling frequency across technologies we created an hexagonal-shaped point grid (HPG) with a predetermined sampling frequency onto which pixel level annotations are mapped. Similar to any filter, choosing a specific spatial resolution will impact the ability to discern fine structures but will also enhance robustness and global features. Therefore, researchers should adjust the sampling frequency to match the spatial variability that is necessary to address their research question.

The OrganAxis model

We define *P* as any point in space, *S* as a spatial domain or tissue region. In our study, *P* is a member of the HPG (**Supplementary Figure 2b**) but this does not necessarily have to be the case, as illustrated in the simulations in **Supplementary Figure 3**. We further establish the sampling frequency (*r*), which refers to the spacing between grid spots within the image, and *K* to be the number of nearest neighbours to be considered for k-nearest neighbour tree (KNN) construction. The minimal distance function denoted as (*D*) calculates the minimal distance from every point *P* to the *K*-nearest points of *S1* and *S2*. We then derive an estimate of minimal distance (μ) from point *P* to the structures *S1* and *S2* by averaging across *D* (**Supplementary Figure 2**, Equation 1). Subsequently, for any point *P* within structures *S1* and *S2*, the relative signed (directional) position with respect to the interface dividing two structures can be computed. This is done through function *H*, which calculates the normalised difference between *μS1* and *μS2* (**Supplementary Figure 2c**, Equation 2). Simulations of H with respect to a linear axis parallel to S1 and S2 produces a sigmoidal-shaped curve, which converges towards -1 and 1, with 0 marking the boundary between structures S1 and S2 (**Supplementary Figure 3a-c**). The established model has two key features: Near the boundary between two regions S1 and S2 (around x = 0) the function H is most sensitive to changes in the value of P (changes in Euclidean position). In

this region, even a small change in P can lead to a relatively large change in output score. This sensitivity is due to the steep slope of the curve at its midpoint. For P that is further away from the boundary, towards either positive or negative infinity, the output of H approaches its maximum (1) or minimum (-1) value, respectively. In these regions, the curve slope is very small, hence changes in P have diminishing effects on the output score.

This behaviour of H function could thus capture biological processes at the boundary between two structures involving the production of biologically active molecules that diffuse from one tissue region, over the boundary to the other, such as cytokines. For instance, within a relatively close proximity of 50 μm to a structure, a positional difference of 20 μm from the source could represent a significant disparity in the level of exposure to a particular cytokine. Conversely, at a larger distance, such as 200 μm away from the source, e.g., a cytokine secreting cell, a similar absolute difference in position becomes negligible in terms of exposure for the receiver cell. Moreover, it may be possible to model different numbers of producing sources of the biologically active molecule by varying the parameter *K* used for minimal distance calculations. This model, of course, constitutes a simplified case and does not account for other means of control of molecule distribution, such as sequestration or shuttling.

<u>Organ Axis hyperparameters: spatial resolution (r) and number of nearest neighbours (K)</u>

To illustrate how the number of nearest neighbours (K) or the grid density (r) influences the transformation of space into axis positions, we conducted simulations using randomly placed spots within an ordered grid (**Supplementary Figure 3a-c**). Our analysis primarily focuses on the relationship between the linear position along the y-axis and the axis function H, in respect to the boundary between two structures.

<u>Changing the *K* nearest neighbours and keeping the grid constant</u>

With K = 2, H exhibits a "noisy" appearance, due to the predominance of values reflecting the simulated grid sparsity. In addition, H exhibits a more step-like function shape. Both of these effects are due to the increased influence of the local environment over the global position. At higher K, e.g., K = 30, the slope of H around the boundary is smaller and typical values are more continuous. At this K, edge effects become apparent where the score is diminished by the lack of grid spots at the edges where P is “exiting” the local environment (**Supplementary Figure 3a,c**) and H thus starts converging towards 0. This property of H is beneficial in cases such as the CMA where several H functions are combined. Spots outside the grid (not simulated here) will rapidly converge to 0 and for P which is not inside S1 or S1 only one H function will have a significant effect.

Changing the grid spacing r and keeping *K* constant

At low grid density of the r relative to K, e.g., r = 5 and K = 10, the transition at the border between the two regions is more gradual and some edge effects are visible (**Supplementary Figure 3b,c**).

These illustrations demonstrate that the choice of r and K should be adjusted to the specific tissue and research question to obtain an optimal level of detail while avoiding artefacts. That said, we emphasise that the shown values of r and K represent extremes, which were chosen for illustrative purposes, and that the axis model is very robust for more moderate changes in r and K

Model derivation for the CMA

We approached the application of OrganAxis to the thymus keeping several biological points in mind:

1. The most abundant cell type in the thymus (thymocytes) is highly migratory and in the process of ongoing differentiation and maturation.
2. The thymus consists of two well-defined anatomical compartments (cortex and medulla).
3. There is a need for a continuous axis to study processes of T cell differentiation and maturation at a resolution beyond the distinction of cortex and medulla.
4. To fully understand the input a particular cell receives, there is a need to account for the global environment (location in a major anatomical structure) as well as the local environment (proximity to the capsule or cortico-medullary junction (CMJ) from both sides of the junction).

To fulfil these requirements, we established the CMA using the following framework:

1. Combination of two axes:

   To better incorporate multiple structures into our model (namely capsule, cortex, and medulla), we designed the CMA as a linear combination of two H functions: Cortico-Medullary (from cortex to medulla) and Edge-Cortical (from capsule/edge to cortex). Given that these functions are signed (directional) and converge to 0 outside S1 and S2 (as described above), we have the flexibility to determine their interplay and the extent to which one influences the other, e.g., specific H could contribute to a “cortical” spot being more “capsular” or more “medullary” by the order of S1(Capsule) and S2(Cortex), which would increase or diminish the final point P score. Furthermore, by weight (w) we can control the extent of that influence to maintain the desired properties

of the axis, like the monotonous increase of the CMA across the entire thymus lobular structure (**Supplementary Figure 2,** Equation 3).

2. K and r choice for the thymus:

   In this study, we selected K = 10 and r = 15 µm, since we found this combination to offer an optimal balance for accurately following the lobular curvature and spatial frequencies in both fetal and paediatric samples, while effectively capturing the intrinsic tissue gradients across different ages while minimising the effects of tissue artefacts. For a detailed visual representation of how adjustments to the K axis parameter influence the analysis of fetal and paediatric image data, see **Supplementary Figure 4a-d**.

3. Axis binning for improved interpretation:

   A fundamental principle of creating a CCF is the development of a standardised language that enables effective communication and knowledge sharing within the scientific community. We are aware that terms like "thymus CMA position 0.5 to 0.6" might not be immediately clear or biologically meaningful and hence further refined the axis into anatomical bins, which correspond to anatomically significant positions and thereby enhance the framework's clarity and utility. This concept is visually demonstrated in **Supplementary Figure 5a-c**. Of note, while the axis is continuous, bins are assigned by cut-off values guided by the need to represent meaningful spatial compartments based on thymus anatomy. Overall, the relative region sizes (number of spots) are similar between fetal and paediatric datasets. Exceptions to this are the capsular region, which is relatively larger in the fetal thymus due to smaller dissociated lobules, and the deep medullary level 3, which is more abundant in paediatric thymus due to more developed medullary regions at this stage (**Supplementary Figure 5e**). Axis bins maintain high accuracy and distinct separation of cortical and medullary regions. This is due to the model's high sensitivity at borders, ensuring that continuity is achieved without compromising the precision of discrete boundaries. The PVS resides either in the medullary CMJ or capsular bins whereas Hassall’s corpuscles (HC) are predominantly found in the deep medullary levels of the paediatric thymus (**Supplementary Figure 5d**).

4. “Horizontal” integration

   The CMA enables direct comparisons of gene and cell distribution patterns across various thymus samples, conditions, technologies, and institutes (**Supplementary Figures 6-11**). Comparing these distributions can also highlight any inconsistencies among samples, guiding researchers to investigate whether these variances stem from technical issues or biological differences. Conversely, when distribution patterns align

across samples, it bolsters confidence in biological insights, surpassing what we can learn from broad, discrete annotations.

Supplementary Note 3: Immune and stromal cell types in the scRNA-seq dataset

Beyond the T lineage, the thymus is home to an abundance of resident hematopoietic cell types (**Supplementary Figure 12a-f**), many of which are directly involved in supporting selection of differentiating thymocytes through the presentation of antigens. In our scRNA-seq dataset we detected a range of B cells, including *TCL1A*$^{+}$ naive, memory B cells and *XBP1*$^{+}$ plasma cells, which all showed the strongest enrichment in the lower levels of the medulla (**Supplementary Figure 12a,b,g**). In the fetal thymus we further annotated proliferating B cells located in the medulla and developing *VPREB1*$^{+}$ B cells at various maturation stages[16], which were less abundant and spatially positioned throughout the cortex and in the CMJ (**Supplementary Figure 12a,b,g**).

In the myeloid compartment we distinguished subsets of conventional DCs (cDC1: *CLEC9A*$^{+}$, cDC2: *CD1C*$^{+}$), three subtypes of activated DCs (*CCR7*$^{+}$ *LAMP3*$^{+}$) first described in our previous study[3], and plasmacytoid DCs (pDCs, *CLEC4C*$^{+}$) (**Supplementary Figure 12c,d**). Proliferating subsets of cDCs and pDCs were detected and most abundant in the fetal thymus. We also identified three subtypes of macrophages: fetal-specific *LYVE1*$^{+}$ macrophages, which have previously been described in other tissues[68,69], a *SPIC1*$^{+}$ thymic subtype observed in mouse[70], and a subtype of *APOC2*$^{+}$ macrophages (**Supplementary Figure 12c,d**). Finally, we noted subsets of developing monocytes and neutrophils mostly derived from the fetal thymus as described in Suo et al.[16] (**Supplementary Figure 12e,f**). In accordance with their involvement in antigen presentation during negative selection, most DC subsets were predicted to predominantly reside in the medulla or around the medullary CMJ of the fetal and paediatric thymus. Paediatric DC1 were an exception to this and also showed some enrichment in cortical and capsular regions. Most DC subtypes in the fetal thymus also showed minor capsular and cortical localisation (**Supplementary Figure 12g**). *SPIC1*$^{+}$ and fetal *LYVE1*$^{+}$ macrophages were predicted to reside in the cortex, while *APOC2*$^{+}$ macrophages were mostly medullary in both fetal and paediatric thymus (**Supplementary Figure 12g**). Most of the monocyte and neutrophil subsets were enriched in capsular and medullary regions (**Supplementary Figure 12g**), pointing to their possible location within blood vessels in those areas.

In addition to TECs, which we describe in detail in **Figure 4**, the thymic stroma harbours a broad range of cell types, including various subsets of fibroblasts, endothelial cells, and

pericytes (**Supplementary Figure 13a-f**). Thymic fibroblasts can broadly be grouped into capsular fibroblasts, which are a source of growth and signalling factors for TECs and T cells, and medullary fibroblasts, which form an interconnected network of conduits in the medulla that sequester cytokines such as CCL21[71]. Some of the medullary fibroblasts are also capable of presenting antigens and thus contribute to negative selection[72]. In our dataset we annotated two sets of capsular fibroblasts, which we refer to as Interlobular ("InterloFb") and Perilobular ("PeriloFb") according to our previous atlas[3] (**Supplementary Figure 13a**). As expected, Visium-based spatial mapping indicated a clear capsular and subcapsular localisation for these two fibroblasts subtypes in both fetal and paediatric thymi (**Supplementary Figure 13g**). In line with the description of medullary fibroblast subsets in recent studies[12,38], we identified three subtypes of putative medullary fibroblasts ("medFb"), which displayed expression of the medullary cytokine *CCL19* as well as the cytokine *IL33* and TNF receptor ligand *TNFSF10* (**Supplementary Figure 13b**). In the paediatric thymus, all medFB subsets were indeed predicted to reside in the medulla. In contrast, in the fetal tissue only medFB-MHCII^{hi} were mapped to this region, while the other two medFB subsets showed cortical enrichment, potentially indicating ongoing migration or maturation of certain fibroblast types in the fetal thymus (**Supplementary Figure 13g**). Finally, we identified rare subsets of fetal-specific fibroblasts ("fetFB") that could not be detected in the paediatric thymus (**Supplementary Figure 13b**). Since these were very lowly abundant, originating from a single donor, and difficult to connect with other thymic fibroblasts, we suspect that these represent a potential contamination from other organs or associated structures, e.g., lymphoma-associated aggregate (fetFB-CCL21) or muscle (fetFB-NKX2-5).

The vascular compartment was represented in our dataset by arterial ("Art", *HEY1*$^+$), venous ("Ven", *PLVAP*$^+$), and capillary cells ("Cap", *RGCC*$^+$) as well as mural cells, including pericytes (*RGS5*$^+$) and smooth muscle cells (*ACTA2*$^+$) (**Supplementary Figure 13c-f**). Most vascular cells, including arterial/capillary endothelial cells, pericytes, and elastin-expressing venous endothelial cells ("EC-Ven-ELN") were predicted to be situated in the capsular/subcapsular regions of the fetal thymus (**Supplementary Figure 13g**). In contrast, only cells of capillaries and lymphatic vessels showed cortical mapping in the paediatric thymus, while arterial cells and most pericytes were enriched at the CMJ or in the medulla. In both age groups, venous cells were mostly detected in the medulla and at the CMJ, together with smooth muscle cells and *CCL19*-expressing pericytes (**Supplementary Figure 13g**).

Consistent with these predicted differences in the distribution of fibroblasts, endothelial cells, and pericytes across development, RareCyte imaging of relevant markers (**Supplementary Figure 14a**) highlighted large structures in fetal thymus consisting of fibroblasts (VIM+) and

blood vessels ($CD31^+$) in the capsular and septal regions (**Supplementary Figure 14b**). These were decreased in paediatric samples, where the largest vessel formations were instead found in the superficial medulla and CMJ (**Supplementary Figure 14b**).

Supplementary Note 4: Annotation of T lineage differentiation stages in fetal and paediatric scRNA-seq data

Through integration of previously published and newly generated scRNA-seq data we were able to establish a dataset containing a total of 76,770 fetal and 277,919 paediatric T lineage cells. We annotated developmental stages based on known marker genes and subsequently used this single-cell reference to deconvolve fetal and paediatric Visium data. Through CMA mapping, we were able to determine the localisation of differentiating thymocytes and compare the local enrichment between fetal and paediatric tissue. Our annotations delineate the classical maturation trajectory (ETP, DN, DP(Q), DP(P), αβT(entry), CD8SP and CD4SP) (**Supplementary Figure 15a**) but within these we noted several subsets with distinct signatures, such as DN(Q)-stress, which expressed *HSPH1* and *JUNB*, and several clusters of DP(Q) thymocytes, which were characterised by the expression of *HSPH1* or *CD99* (**Supplementary Figure 15b**) or predicted by Dandelion[47] to be associated with late V(D)J pseudotime based on TCR-seq results. Since these cell subsets have not been described before and we cannot exclude that these represent technical artefacts of tissue dissociation, we did not explore these further. We also annotated several subtypes of Tregs, CD8αα thymocytes, γδ T cells, and NK cells. Comparison of marker gene expression profiles across the annotated stages demonstrated these cell types to be highly comparable between fetal and paediatric data (**Supplementary Figure 15b**), confirming the robustness of our annotations. We explored the localisation and the canonical T cell maturation trajectory in **Figure 3** and showed that this is very similar between fetal and paediatric thymus. Likewise, additionally annotated T lineage subtypes were enriched in similar thymic regions independent of age, for instance, in both fetal and postnatal thymus CD8αα(entry) thymocytes were detected in the cortex, whereas CD8αα(I) and CD8αα(II) were predominantly enriched in the upper and lower medullary levels, respectively (**Supplementary Figure 15c**). Tregs were exclusively detected in the medulla, independent of their subtype, as expected based on their differentiation following medullary negative selection.

Note that we subsequently established a high-resolution annotation of developing T cells using complementary surface marker and transcriptome profiling via CITE-seq. Please refer to **Figure 6** and **Supplementary Note 6** for the relevant findings.

Supplementary Note 5: Localisation of paediatric mcTECs is associated with differential priming towards cTECI vs. mTECI fate

Our Visium and IBEX analyses of paediatric TECs revealed two distinct sites of mcTEC enrichment, the capsular region and the CMJ. In order to investigate potential localisation-associated differences between the two mcTEC subsets, we conducted fate prediction analysis on the scRNA-seq data using STEMNET[50]. We defined cTECI/II/III and mTECI/II/III as maturation endpoints and determined the potential for all cTEC, mTEC, and mcTEC cells to differentiate into these six TEC subtypes. STEMNET visualises this by placing the maturation endpoints on the corners of a simplex and arranges individual cells according to their predicted priming towards each of the six possible fates (**Supplementary Figure 16a,b**). mcTECs were predicted to be primed towards cTECI or mTECI fate in varying degrees, but showed no notable priming towards cTECII/III or mTECII/III, while mcTEC-Prolif were almost exclusively primed towards cTECI fate. We calculated the difference for the predicted cTECI vs. mTECI potential, which yielded a priming score between -1 (fully mTECI-primed) and 1 (fully cTECI-primed). Cells were labelled as “cTECI-primed” if their score was above 0.5 and “mTECI-primed” if their score was below -0.5, while all other mcTECs were labelled “unprimed” (**Supplementary Figure 16c,d**). scRNA-seq gene expression analysis showed that cTECI-primed mcTECs expressed higher levels of the cortical marker *LY75*, stem cell marker *ITGA6* as well as *NFATC2* and *KLK2*, while mTECI-primed mcTECs expressed higher levels of *IGFBP6*. Importantly, all mcTECs were clearly distinct from mature mTECs and cTECs based on their lack of *HLA-DPB1* expression (**Supplementary Figure 16e**). We then mapped the newly-defined mcTEC subtypes back to the paediatric Visium data, which revealed enrichment of cTECI-primed mcTECs in the capsular region, while mTECI-primed and unprimed cells were predominantly found in the medulla and around the medullary CMJ (**Supplementary Figure 16f,g**). To further validate these findings, we utilised segmented IBEX nuclei data with annotations obtained via KNN mapping and separated IBEX mcTECs into capsular or CMJ subsets based on their CMA-derived localisation (**Supplementary Figure 16h**). Importantly, protein information from IBEX data confirmed higher expression of CD205 (corresponding to *LY75*) and CD49F (corresponding to *ITGA6*) in capsular mcTECs, while CMJ mcTECs were higher in KRT15. mcTECs in both locations were low in MHC-class II proteins and demonstrated high levels of LAMIN A expression. (**Supplementary Figure 16i**). In summary, these findings suggest that mcTECs in the capsular regions exhibit preferential priming for the cTECI fate, express cortical marker CD205/*LY75* and stem cell marker CD49F/*ITGA6*, whereas the CMJ harbours mcTECs that are either unprimed or mTECI-primed and have higher levels of KRT15 protein and *IGFBP6* transcripts.

Supplementary Note 6: CITE-seq permits fine grained annotation and high-resolution spatial mapping of the T lineage

To profile T cell development in the human postnatal thymus at increased resolution, we generated a multimodal dataset by performing CITE-seq, which enables paired quantification of transcripts and surface protein levels at single-cell resolution. We employed a 143-plex customised TotalSeq-C antibody panel and combined this with scTCR-seq for the αβTCR to obtain information about TCR rearrangements. The RNA and surface protein modalities were subsequently integrated using the Weighted Nearest Neighbours (WNN) approach[58] and cell types and maturation stages were annotated based on their surface markers and gene expression.

For the annotation of the earliest thymocyte differentiation stages, we relied on the expression of the commonly used surface markers CD1A, CD34, CD44, CD4, and CD8[4,73] and annotated cells before ("uncommitted", $CD34^{hi}$ $CD1A^{-}$ $CD44^{+}$) and after T lineage commitment ("committed CD4neg", $CD34^{+}$ $CD1A^{+}$ $CD44^{-}$ $CD4^{-}$) as well as immature single positive (ISP) thymocytes ("committed CD4pos", $CD34^{+}$ $CD1A^{+}$ $CD44^{-}$ $CD4^{+}$ $CD8^{-}$). Cell scoring for cell cycle-associated genes indicated proliferating (P) subsets within these stages. We further detected a population of quiescent DP thymocytes that clustered with the more immature stages and was characterised by a low frequency of *TRB* rearrangements and CD31 expression (**Supplementary Figure 17a,b**), resembling a previously described $CD31^{hi}$ $CD4^{+}$ $CD8\alpha^{+}$ $CD8\beta^{-}$ stage that has been associated with TCR β-selection[74]. All of these immature thymocyte subsets were determined to be predominantly resident in the capsule, subcapsular regions and the outer cortex of the paediatric thymus (**Supplementary Figure 17c**). In addition, uncommitted thymocytes also showed notable enrichment in the upper layers of the medulla, which aligns with the mapping of ETPs to the medullary layers as seen in our scRNA-seq based annotations (**Figure 3e**).

Beyond proliferating and quiescent/*TRA*-rearranging DP thymocytes, we were further able to resolve cells that had undergone positive selection ($CD69^{+}$, $RAG1/2^{lo}$, high proportion of rearranged *TRA*) (**Supplementary Figure 17a,b**), which was followed by a $CD4^{hi}$ $CD8^{lo}$ stage, during which cells have already received the signalling cues instructing their lineage fate but do not yet exhibit a clear lineage-specific transcriptional programme[75,76] (**Supplementary Figure 17b**). Both of these post-selection stages were predicted to still be localised in the cortex but already showed a shift in their spatial distribution towards the CMJ (**Supplementary Figure 17c**). Following the lineage bifurcation, $CD4^{+}$ and $CD8^{+}$ SP thymocytes could be distinguished in their maturation progress based on commonly used markers, such as CD1A, CD69, CD27, CD45RO, and CD45RA[4] (**Supplementary Figure

17a). Spatial mapping indicated a change in localisation to the thymic medulla for all subsets except immature CD8SP thymocytes, which were still mainly detected in the cortex (**Figure 6a, Supplementary Figure 17c**). The CITE-seq profiling was critical for the distinction of several Treg stages and subsets, such as $CD103^+$ $CD8^+$ Tregs[77] as well as recirculating Tregs[78] ("Treg_recirc", $CD39^+$ $CD31^-$) (**Supplementary Figure 18a**), all of which were predicted to reside in the medulla (**Supplementary Figure 18b**). We were further able to annotate several unconventional T cell subsets, such as CD8αα(I) and CD8αα(II) cells, as well as differentiating γδ T cells, with immature cells mapping to the cortex/CMJ, and mature cells found in the medulla (**Supplementary Figure 18a,b**). Lastly, we detected a limited number of circulating ($CD62L^{hi}$) and tissue resident ($CD69^{hi}$) NK cells[79], which could be separated by CD56/CD16 expression or integrin levels (IntegrinB7, CD103, CD49a), respectively. We also noted a small subset of DP cells with NK phenotype ($CD16^+$ $CD56^+$ $CD161^+$) and ongoing *TRA* rearrangements, which most likely correspond to developing NKT cells. All NK cell subsets were predicted to reside in the thymic medulla whereas the putative developing NKT cells mapped closer to the CMJ in accordance with their DP phenotype and origin (**Supplementary Figure 17b, 18a,b**).

Supplementary Discussion

Large scale efforts of international consortia like the HCA[80] and HuBMAP[66] have emphasised the need for constructing extensive tissue atlases to offer a basis for deeper understanding of cell and human biology. Initially consisting of analyses of dissociated cells, next generation spatial atlases now provide crucial insights into cell interactions and the structural underpinnings of complex tissues and organs. However, harmonisation of spatial datasets remains a challenge and represents a major hurdle for multi-group consortia. Here, we offer a new integrative computational approach for spatial data analysis and implement these novel tools for both spatial transcriptomics and multiplex protein imaging methods. Combined with newly generated multimodal single-cell data and previously published datasets, we have created a thymus spatial atlas spanning fetal and early paediatric life that was curated specifically to cover developing T cells as well as thymic hematopoietic and stromal cells at higher resolution and diversity than previously achieved.

By conducting CITE-seq and scTCR-seq we have gathered whole transcriptome readouts, protein expression of 143 cell surface markers and TCR rearrangement status for over 30 stages of conventional and unconventional T cell development in the human paediatric thymus. This trimodal dataset can serve as a critical bridge between classic methodological approaches and recently developed methods, e.g., antibody-based applications, such as flow cytometry or immunofluorescence imaging, and nucleic acid-based approaches, like scRNA-seq or spatial transcriptomics, through imputation of the missing modality. We further integrated a large cohort of fetal and early paediatric Visium spatial transcriptomics data and an extensive paediatric IBEX 44-plex imaging dataset based on a custom TEC- and T cell-focused antibody panel to chart both micro- and macroscopic tissue architecture and spatial cellular compositions. Finally, we provide a comprehensive RareCyte multiplex imaging dataset that we used for validation but will hopefully serve as a basis for future analysis. These data can be used to directly compare protein and RNA for cell types in their local environment as well as larger structural anatomical features.

Beyond these reference datasets, we have developed an extensive computational tool set for annotation, processing, and analysis of imaging and spatial transcriptomics data (TissueTag). Moreover, we have established the OrganAxis model, which can serve as a CCF for the integration of spatial data derived from morphologically diverse samples profiled with different approaches. In this study, we adapted the OrganAxis framework to the thymus context to establish the CMA, which we used to obtain a holistic representation of the human thymus throughout early development. This key immunological organ is highly dynamic and can undergo rapid macro-morphological changes within the course of days, e.g., during

infection, as well as more gradually during ageing[81,82]. Moreover, the vast majority of thymic cells are T cells at various stages of development, which are constantly moving throughout their entire maturation process. Changes to the relative size of various classically defined compartments within the organ as well as the migration patterns of the constituent cells make it difficult to determine the anatomical location of cell types and distinguish random from consistent spatial associations. By applying the CMA to the spatial data, we quantify local cell abundances within thymic substructures and establish changes in the spatial composition of the developing thymus at unprecedented resolution. We discovered that spatial patterns of T cell maturation stages, major TEC subtypes, and critical chemokine gradients are already established by the beginning of the second trimester and remain largely unchanged in early paediatric thymus. This complements our previous single-cell atlas[3], which indicated a stabilisation of the thymic cellular composition around this fetal developmental time point. At this age the thymic architecture is still not fully established, and differentiation of TEC subsets is still ongoing[1,11]. In contrast, the mouse thymus only starts to develop in mid-gestation, produces the first SP thymocytes at E16[83], and still increases in size and cellularity up to one month after birth[84]. Importantly, a comparison between fetal and paediatric human thymus revealed differences in the tissue niche of TEC progenitors ("mcTECs") between the two age groups. We show that in the human postnatal thymus these cells are located both in the capsular region and around the CMJ/PVS and can be primed towards cTEC or mTEC fate depending on the location. This aligns with mouse studies that have suggested postnatal TEC progenitors to be bi-potent and located at the CMJ[24,29,85]. In contrast, we found that human fetal mcTECs were exclusively found in the (sub-)capsular region, which was also highly vascularised, abundant in fibroblasts and hematopoietic cells, and characterised by expression of certain chemokines and may thus represent the primary area of organ growth. Capsular regions undergo substantial thinning throughout development and especially after birth, which indicates re-organisation of this region in the paediatric thymus.

In addition to studying cellular gradients along the CMA, we utilised annotation of HCs to explore proximal cell associations with this important medullary structure. This confirmed association of mTECIII with HCs and highlighted their further specialisation into sub-lineages expressing keratinocyte (mTECIII-skin) and mucosal epithelia genes (mTECIII-muc), which likely mimic peripheral tissue antigens. Importantly, mTECI-III exhibited gradient-like organisation increasing in abundance towards the deep medulla and around HCs, with mTECI being the most diffuse, followed by mTECII and mTECIII almost exclusively concentrated around HCs. This might hint at how negative selection occurs in an efficient manner by promoting central tolerance through more spread out mTECII expressing various

tissue antigens in a stochastic manner[86] and highly localised mimetic cells (mTECIII, TEC-neuro, TEC-myo) expressing groups of genes associated with particular tissues[6].

Finally, fine-grained annotation of the T lineage in combination with CMA mapping and trajectory analysis allowed us to establish the cortico-medullary migration kinetics of the late stages of thymocyte maturation at high resolution. While intrathymic migration patterns have been studied on a smaller scale using *in vitro* live imaging[41,87], our approach has the advantage of linking differentiation progress and migratory activity. This revealed a temporary cortical retention of immature CD8 lineage thymocytes relative to their CD4 lineage counterparts in both Visium and IBEX data. This also illustrates how multimodal datasets can validate and expand the interpretation of a cell's maturation state, its local environment, and its position in a tissue. Importantly, the migratory differences were associated with the expression of different chemokine receptors on the two lineages, which could implicate different stimuli/ligands, potentially derived from different medullary cell types, in promoting medullary entry of $CD4^+$ vs. $CD8^+$ SP thymocytes. Our findings directly align with two recent studies that suggest a slower maturation of CD8 lineage cells[41,88]. Moreover, our observation that co-receptor reversal in CD8 lineage cells coincides with the cortical retention window further indicates that CD8SP thymocytes may require more time to initiate the lineage-specific differentiation programme, which in turn could impact the timing of intercompartmental cell migration. Additionally, the cortex may provide the specific microenvironment and signalling cues that CD8 lineage cells need to initiate their maturation. Future application of this spatio-temporal mapping approach on next-generation spatial methods, such as VisiumHD, will allow the investigation of high-resolution cell-cell communication circuits that is at present still restricted to tailored functional assays.

Overall, we report here a large collection of reference data for the fetal and paediatric human thymus and demonstrate that these data both reconcile prior findings while also leading to a series of novel biological insights in the areas of stromal cell organisation and T cell maturation and migration. We expect that beyond the observations we detail here, there is still much to uncover using these datasets. We have further illustrated the usefulness and applicability of the CMA and hope that this CCF can support future thymus studies by offering a common language of reference for the community, especially when discussing hard to define structures like the CMJ and subcapsular region. Finally, the computational tools and specifically OrganAxis translates well to other organ systems[89] in 2D, 3D and even 4D (time), enabling accurate spatial modelling and CCF construction and providing valuable resources to the broad scientific community.

Supplementary References

63. Simonson, P. D., Ren, X. & Fromm, J. R. Creating Virtual Hematoxylin and Eosin Images using Samples Imaged on a Commercial CODEX Platform. *J. Pathol. Inform.* **12**, 52 (2021).

64. Lancaster, J. L. *et al.* Bias between MNI and Talairach coordinates analyzed using the ICBM-152 brain template. *Hum. Brain Mapp.* **28**, 1194–1205 (2007).

65. Weber, G. M., Ju, Y. & Börner, K. Considerations for Using the Vasculature as a Coordinate System to Map All the Cells in the Human Body. *Front Cardiovasc Med* **7**, 29 (2020).

66. HuBMAP Consortium. The human body at cellular resolution: the NIH Human Biomolecular Atlas Program. *Nature* **574**, 187–192 (2019).

67. Burger, A. *et al.* Towards a clinically-based common coordinate framework for the human gut cell atlas: the gut models. *BMC Med. Inform. Decis. Mak.* **23**, 36 (2023).

68. Chakarov, S. *et al.* Two distinct interstitial macrophage populations coexist across tissues in specific subtissular niches. *Science* **363**, eaau0964 (2019).

69. Eraslan, G. *et al.* Single-nucleus cross-tissue molecular reference maps toward understanding disease gene function. *Science* **376**, eabl4290 (2022).

70. Zhou, T.-A. *et al.* Thymic macrophages consist of two populations with distinct localization and origin. *eLife* **11**, e75148 (2022).

71. Fuertbauer, E. *et al.* Thymic medullar conduits-associated podoplanin promotes natural regulatory T cells. *Immunol. Lett.* **154**, 31–41 (2013).

72. Nitta, T. *et al.* Fibroblasts as a source of self-antigens for central immune tolerance. *Nat. Immunol.* **21**, 1172–1180 (2020).

73. Canté-Barrett, K. *et al.* Loss of CD44dim Expression from Early Progenitor Cells Marks T-Cell Lineage Commitment in the Human Thymus. *Front. Immunol.* **8**, 32 (2017).

74. Douaisi, M. *et al.* CD31, a Valuable Marker to Identify Early and Late Stages of T Cell Differentiation in the Human Thymus. *J. Immunol.* **198**, 2310–2319 (2017).

75. Yasutomo, K., Doyle, C., Miele, L., Fuchs, C. & Germain, R. N. The duration of antigen receptor signalling determines CD4+ versus CD8+ T-cell lineage fate. *Nature* **404**, 506–510 (2000).

76. Lucas, B. & Germain, R. N. Unexpectedly complex regulation of CD4/CD8 coreceptor expression supports a revised model for CD4+CD8+ thymocyte differentiation. *Immunity* **5**, 461–477 (1996).

77. Nunes-Cabaço, H., Caramalho, I., Sepúlveda, N. & Sousa, A. E. Differentiation of human thymic regulatory T cells at the double positive stage. *Eur. J. Immunol.* **41**, 3604–3614 (2011).

78. Morgana, F. *et al.* Single-Cell Transcriptomics Reveals Discrete Steps in Regulatory T Cell Development in the Human Thymus. *J. Immunol.* **208**, 384–395 (2022).

79. Freud, A. G., Mundy-Bosse, B. L., Yu, J. & Caligiuri, M. A. The Broad Spectrum of Human Natural Killer Cell Diversity. *Immunity* **47**, 820–833 (2017).

80. Regev, A. *et al.* Science Forum: The Human Cell Atlas. *eLife* **6**, e27041 (2017).

81. Luo, M., Xu, L., Qian, Z. & Sun, X. Infection-Associated Thymic Atrophy. *Front. Immunol.* **12**, 652538 (2021).

82. Liang, Z., Dong, X., Zhang, Z., Zhang, Q. & Zhao, Y. Age-related thymic involution: Mechanisms and functional impact. *Aging Cell* **21**, e13671 (2022).

83. Zaharie, D., Moleriu, R. D. & Mic, F. A. Modeling the development of the post-natal mouse thymus in the absence of bone marrow progenitors. *Sci. Rep.* **6**, 36159 (2016).

84. Baran-Gale, J. *et al.* Ageing compromises mouse thymus function and remodels epithelial cell differentiation. *Elife* **9**, e56221 (2020).

85. Ishikawa, T., Akiyama, N. & Akiyama, T. In Pursuit of Adult Progenitors of Thymic Epithelial Cells. *Front. Immunol.* **12**, 621824 (2021).

86. Dhalla, F. *et al.* Biologically indeterminate yet ordered promiscuous gene expression in single medullary thymic epithelial cells. *EMBO J.* **39**, e101828 (2020).

87. Witt, C. M., Raychaudhuri, S., Schaefer, B., Chakraborty, A. K. & Robey, E. A. Directed migration of positively selected thymocytes visualized in real time. *PLoS Biol.* **3**, e160 (2005).

88. Steier, Z. *et al.* Single-cell multiomic analysis of thymocyte development reveals drivers of CD4+ T cell and CD8+ T cell lineage commitment. *Nat. Immunol.* **24**, 1579–1590 (2023).

89. Cranley, J. *et al.* Multiomic analysis reveals developmental dynamics of the human heart in health and disease. *bioRxiv* (2024) doi:10.1101/2024.04.29.591736.

Supplementary Figures

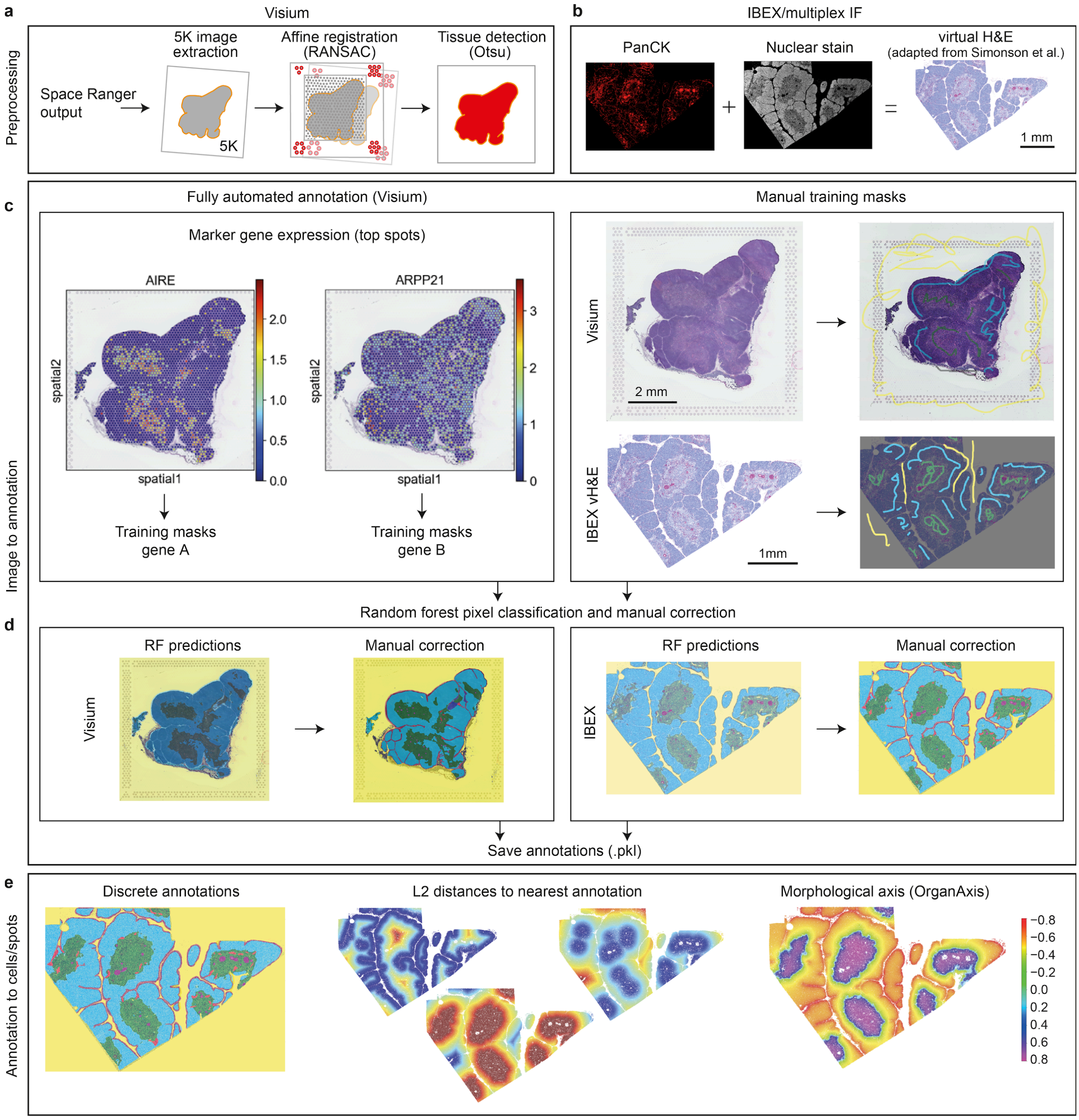

Supplementary Figure 1. Obtaining unified tissue annotations across platforms with TissueTag. a. TissueTag workflow for generating a high-resolution H&E reference image, alignment to the fiducial frame by fiducial detection and Random Sample Consensus (RANSAC), and tissue region detection by the Otsu threshold method. **b.** TissueTag IBEX pre-processing generates a virtual H&E based on two user-defined fluorescent channels, e.g., Pan-Cytokeratin (PanCK, red) and Hoechst nuclear stain (grey). **c.** Image annotation can be achieved by a pixel random forest (RF) classifier, which is trained either by RNA marker genes (*AIRE* for medulla, *ARPP21* for cortex)(left) or manual "scribbling" of tissue annotations on easy-to-call regions (right). **d.** Pixel RF predictions can be corrected and additional fine annotations are added manually. All annotation metadata (region names, annotation resolution, annotation colours) are saved as .pkl files for easy recovery. **e.** Discrete tissue annotations produced with TissueTag are the basis for L2 distance measurements - the minimal Euclidean distance of a spot in space to a discrete structure (e.g., cortex or medulla). These annotations are used to calculate a morphological axis (OrganAxis, see **Supplementary Note 2**).

Definition:

Hexagonal point grid (HPG) is a labelled hexagonal array of spots

r - Spot-to-spot distance of the HPG.

$d_S(p)$ is defined as the euclidean distances between point p,
and all points that belong to structure S

$S \in \{S1, S2\}$

$p \in \Re^2$, points are arranged as a grid with spacing (resolution) r

$D_{S,p}[i]$ is the sorted (increasing) series of $d_S(p)$, where i is the index of $D_{S,p}$

$D_{S,p}[i] \leq D_{S,p}[i+1]$, $\forall$ i

Example: $D_{S,p}[0]$ is the distance to the nearest point in structure S to point p.

Equation 1:

$$\mu_K^S(p) = \sum_{i=0}^{K-1} \frac{D_{S,p}[i]}{K}$$

$\mu_K^S(p)$ is the mean minimal distance, where K is the number of nearest neighbours

Equation 2:

$$H_K^{S1,S2}(p) = \frac{\mu_K^{S1}(p) - \mu_K^{S2}(p)}{\mu_K^{S1}(p) + \mu_K^{S2}(p)}$$

$H_K^{S1,S2}(p)$ defines the position of p relative to the boundary between $S1$ and $S2$

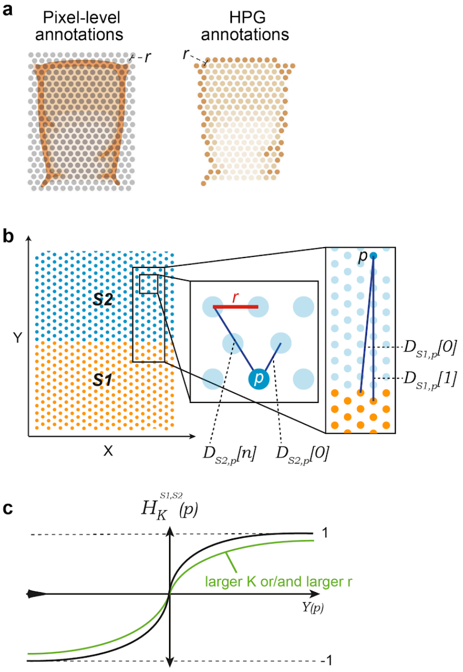

Intuition:

H is a sigmoid-like function between -1 and 1 where 0 is the boundary between $S1$ and $S2$.

(1) If p is inside or very close to $S1$ and far from $S2$, it tends towards the value (-1)

(2) If p is inside or very close to $S2$ and far from $S1$, it tends towards the value (1)

The slope angle and min/max values of H depends on the size of K and grid density:

(1) When K is large (accounting for more spots of S) the transition from the boundary to the edge is more gradual and vice versa

(2) When the grid density is low (large r), the minimal value of H would be large and thus transition along the boundary is more gradual and vice versa

Equation 3 (CMA version):

$w1$ = 0.8, $w2$ = 0.2

$S11$ = cortex, $S12$ = medulla, $S21$ = capsule, $S22$ = cortex

$$\mathrm{CMA}_K(p) = w_1 H_K^{cortex\text{-}medulla}(p) + w_2 H_K^{capsule\text{-}cortex}(p)$$

Equation 4 (general):

$$\mathcal{E}(p) = \sum_j w_j H_{K_j}^{S_{j,1}\text{-}S_{j,2}}(p)$$,where j is an index

Note:

K_j can change between instances of H to factor in more or less of the local neighbourhood versus the global position relative to structures $S1$ and $S2$.

Supplementary Figure 2. Mathematical formulation of the OrganAxis CCF model. Definitions of the mathematical components and equations used for axis calculations. **a.** An illustration of sampling annotations at low resolution. HPG: Hexagonal point grid. **b.** Illustration of grid distance r, distance function D and how the distance from point P to structures S1 and S2 is calculated. **c.** Illustration of the S-like shape of function H and the effect of modifying r or K.

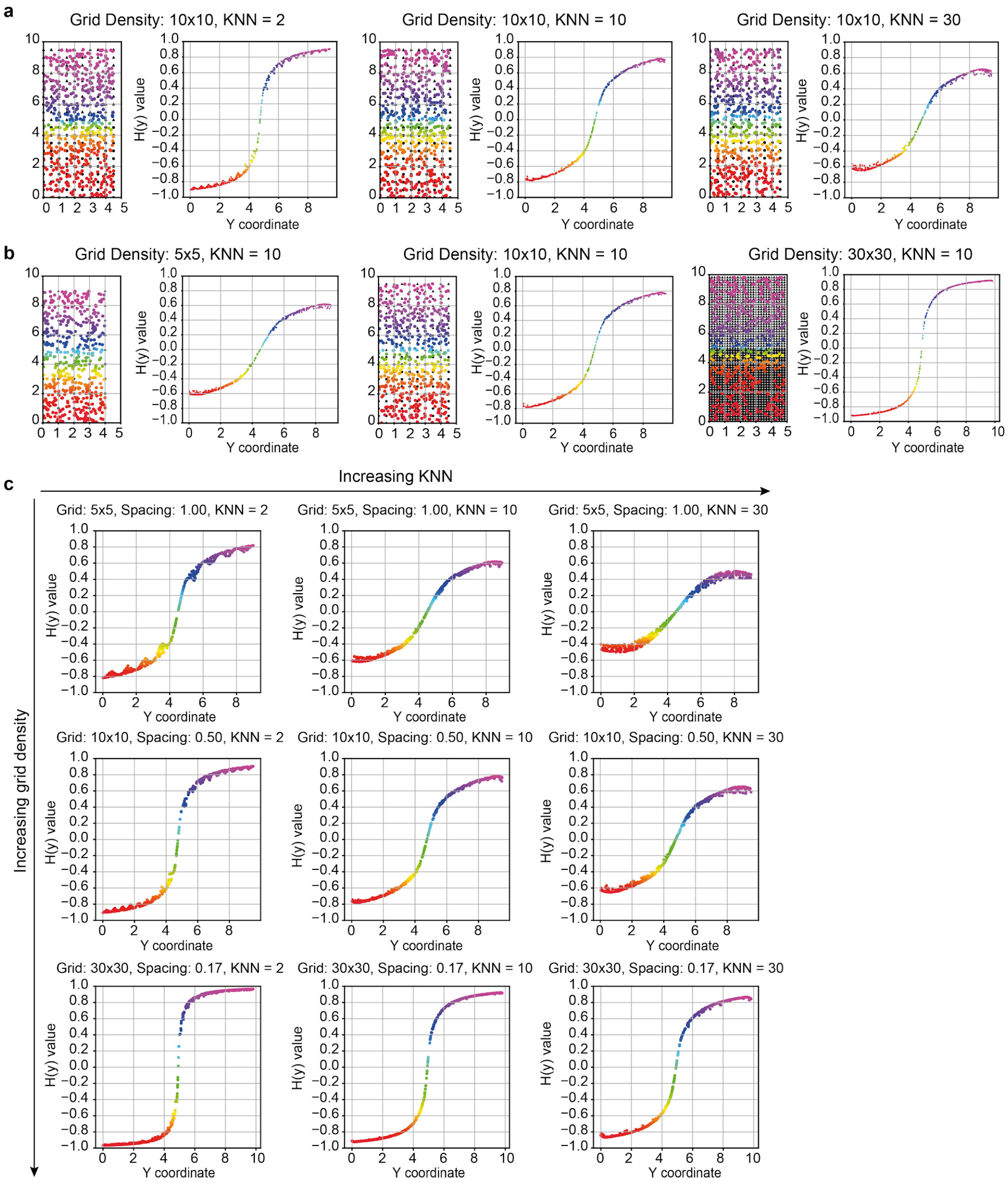

Supplementary Figure 3. **H position function simulations with varying K and r parameters. a-b.** Plots of simulated data (left) to illustrate how random spots in space are assigned a position score H(x) within a space that is made up of two regions, represented by a grid of circles (lower region) and triangles (upper area) and coloured by the score for function H. Plot of score H as a function of y-axis (right). The number of random spots is constant. **a.** For KNN = 2, H is “jagged” due to overrepresentation of local spacing of the simulated grid pattern. This also leads to a more step-function shape since the local environment is overrepresented over the global position. For higher K (KNN = 30), the slope of H around the boundary is smaller and typical values are more continuous. Edge effects become apparent where the score is diminished by a lack of sufficient grid spots for points near the edges, thus effectively increasing the average distance to the grid K spots. **b.** Changing the grid spacing r and keeping K constant: At low grid density r relative to K, the transition at the border between the two regions is more gradual, some edge effects are visible. **c.** Matrix comparison for a combination of r and K parameters.

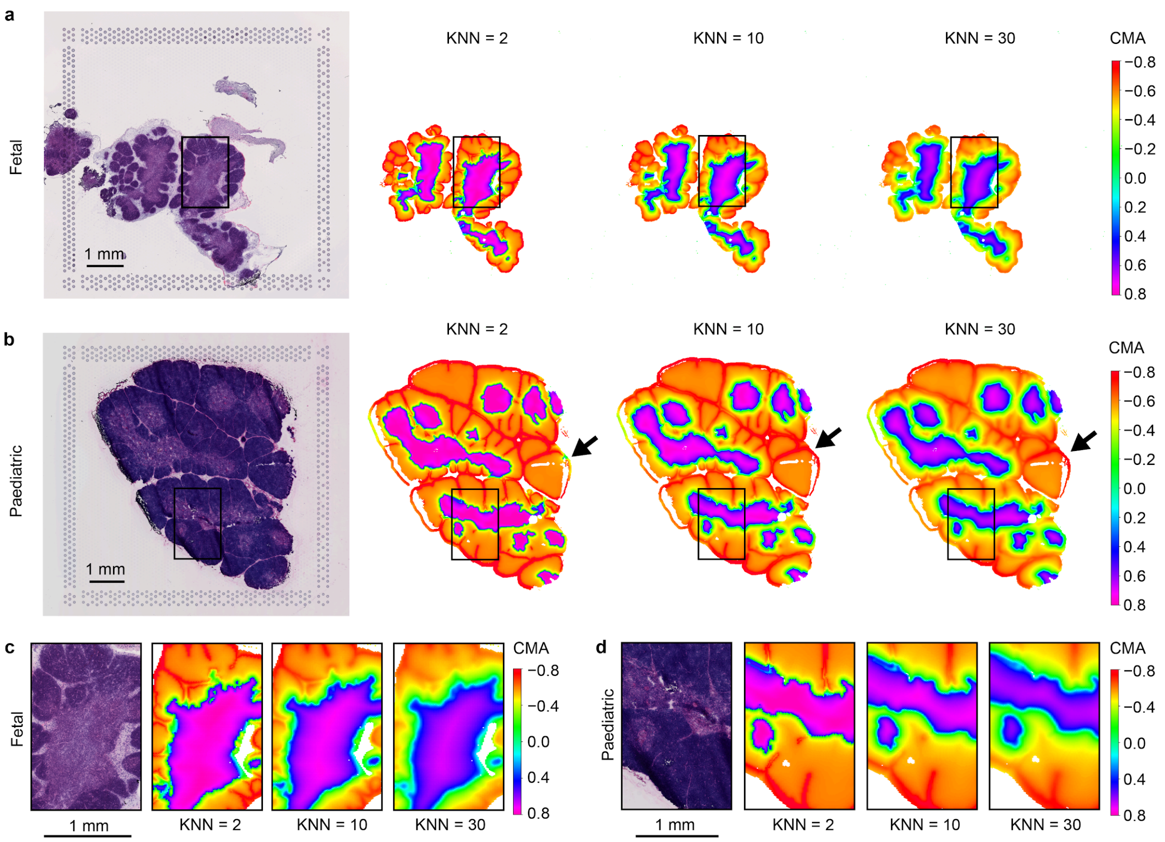

Supplementary Figure 4. Visualisation of the impact of changing the KNN axis parameter on fetal and paediatric image data. a-b. Visium H&E image (left) and the corresponding CMA plots generated with increasing KNN values (right). In the present study, KNN = 10 was used as this reflected an ideal balance between following the curvature of structures for both fetal and paediatric samples while robustly capturing the intrinsic gradients of the tissue across ages. For all plots a HPG with r = 15 μm was used. **a.** Representative tissue section from the fetal thymus (p.c.w. 15). Black frame indicates the region shown in c. **b.** Representative tissue section from the paediatric thymus (3 months old). Black frame indicates the region shown in d. Arrow highlights a region where a few pixels were wrongly annotated at a low value for K. Note how this effect is diminished with larger K values. **c-d.** High-magnification cropped images highlighting how the various K values reflect a balance between local (borders between lobules) and global representation (medullary depth). **c.** Cropped image of fetal thymus for the region indicated in a. **d.** Cropped image for paediatric thymus for the region indicated in b.

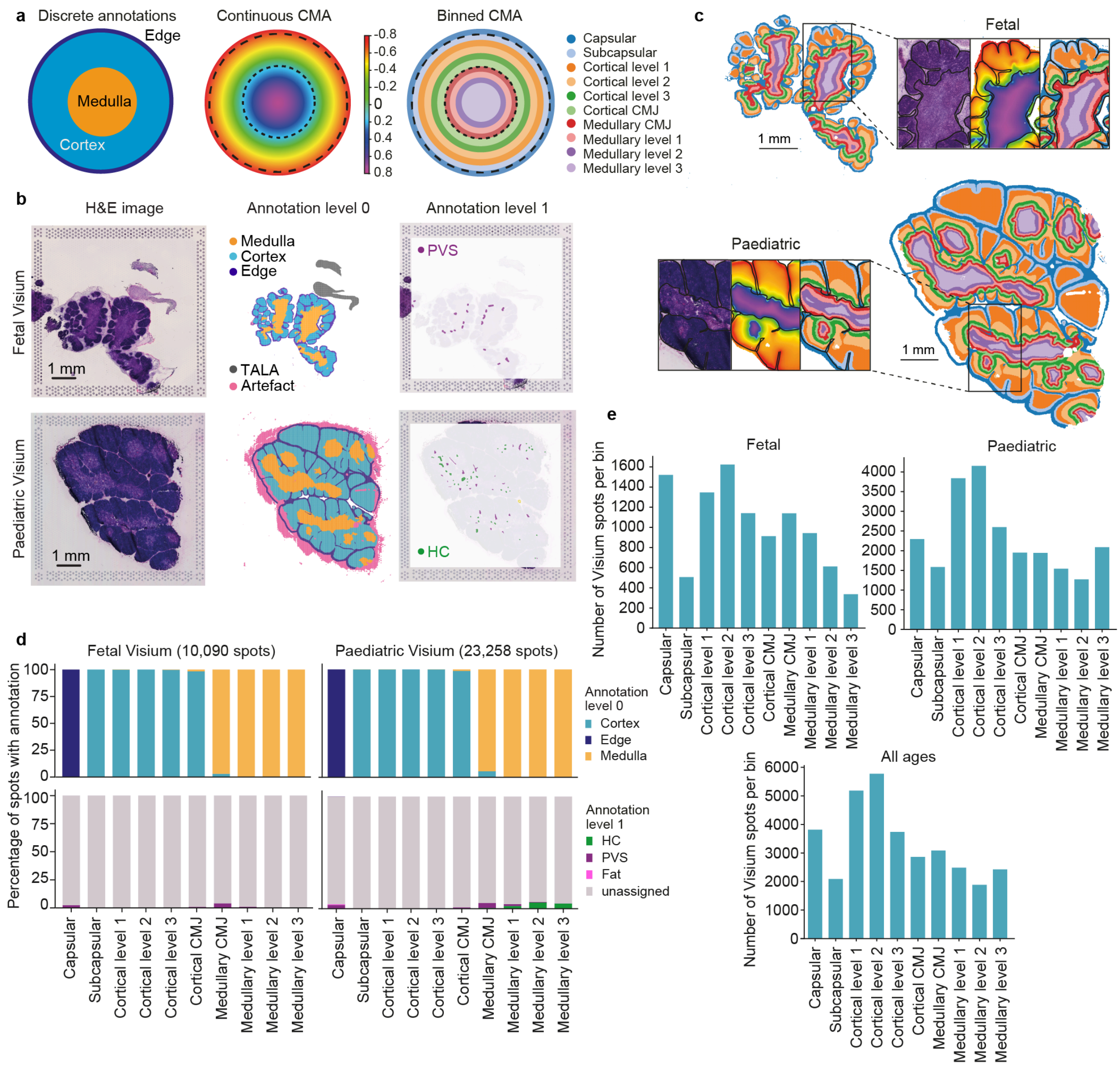

Supplementary Figure 5. Generation of a binned axis. a. Simplified thymus representation depicting a progression from broad anatomical annotations (left) to the continuous axis (centre), and finally to the discretely binned axis (right). **b.** Representative H&E images from fetal (p.c.w. 15) and paediatric (3 months old) Visium sections with the corresponding TissueTag annotations of broad regions (annotation level 0, centre) and fine structures (annotation level 1, right). TALA: Thymus-associated lymphoid tissue. **c.** Binned CMA space on fetal (p.c.w. 15) and paediatric (3 months old) Visium sections with magnified regions showing H&E, continuous CMA, and binned CMA (from left to right). Black lines indicate cortical and medullary regions for comparison across the three representations. **d.** Spot composition of CMA bins by discrete annotations of the two annotation levels shown in b. Note that discrete annotation separation is preserved. **e.** Spot counts for Visium datasets in fetal, paediatric, and combined spots. Note that aside from capsular and sub-capsular spots, which were determined to capture specific structures, the frequency of Visium spots diminishes gradually with CMA value as is expected by equidistant sampling of a spherical structure as depicted in a.

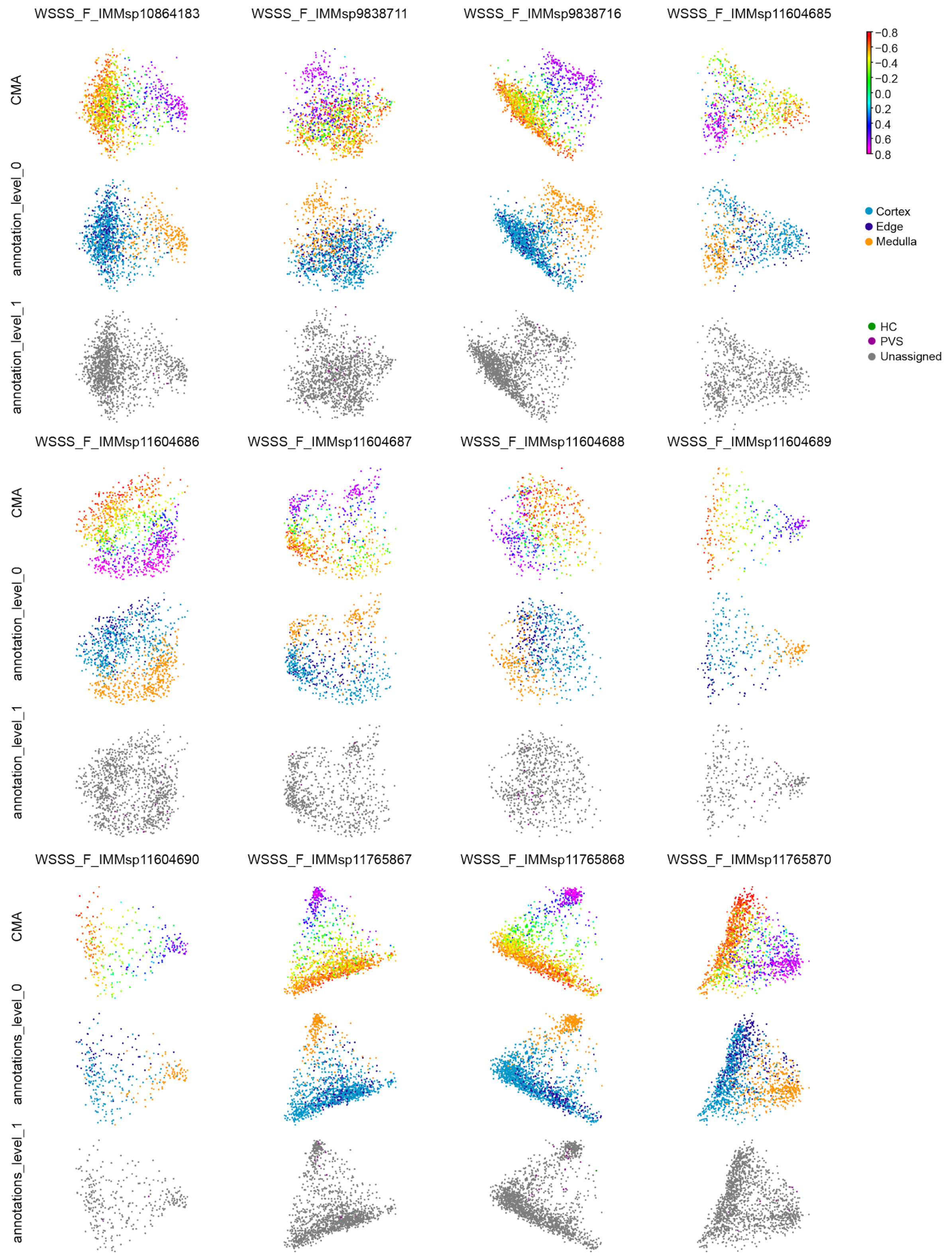

Supplementary Figure 6. PCA plots for 12 individual fetal Visium samples highlighting the major tissue annotations: CMA, annotation level 0 and annotation level 1.

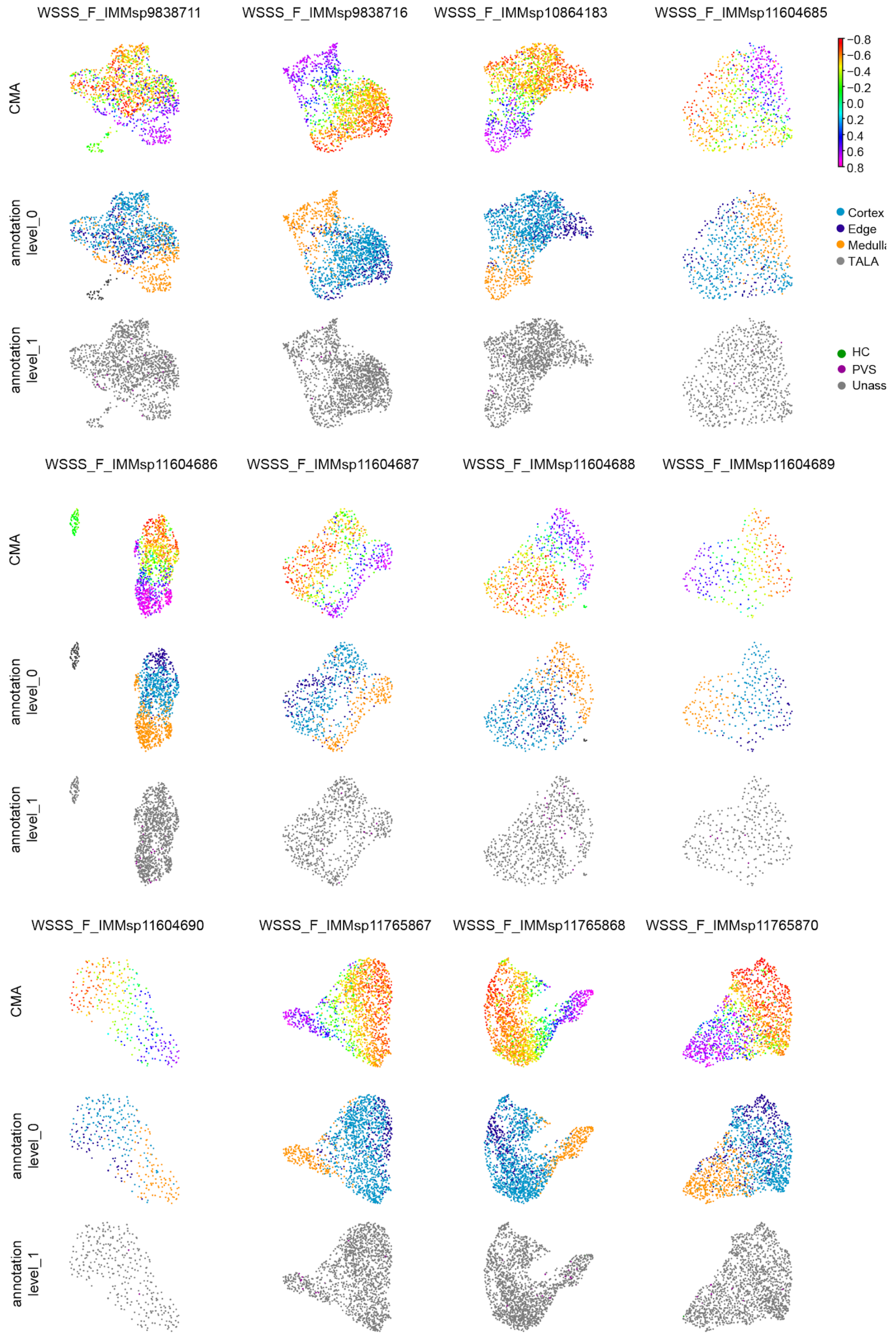

Supplementary Figure 7. UMAP plots for 12 individual fetal Visium samples highlighting the major tissue annotations: CMA, annotation level 0, and annotation level 1.

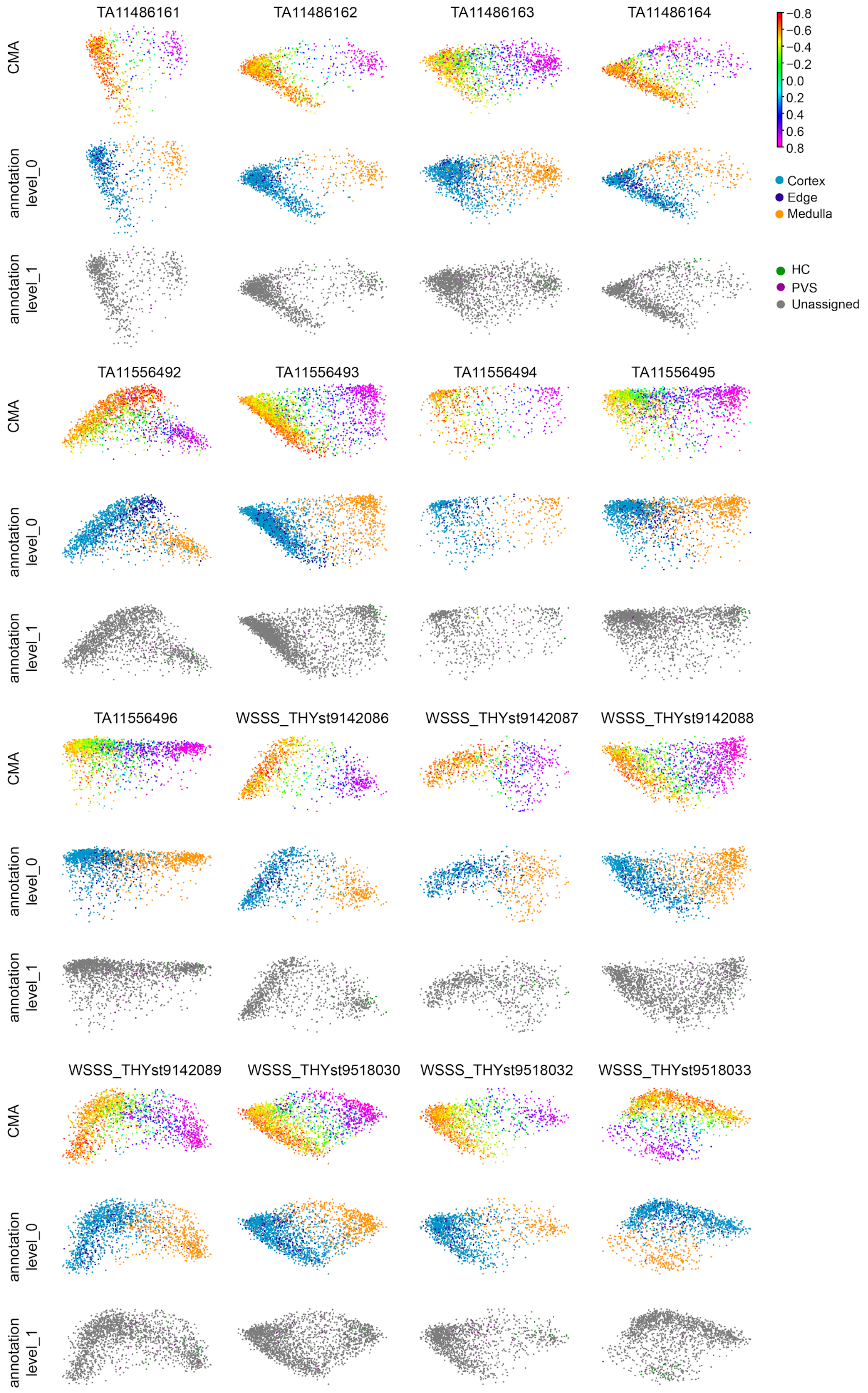

Supplementary Figure 8. PCA plots for 16 individual paediatric Visium samples highlighting the major tissue annotations: CMA, annotation level 0, and annotation level 1.

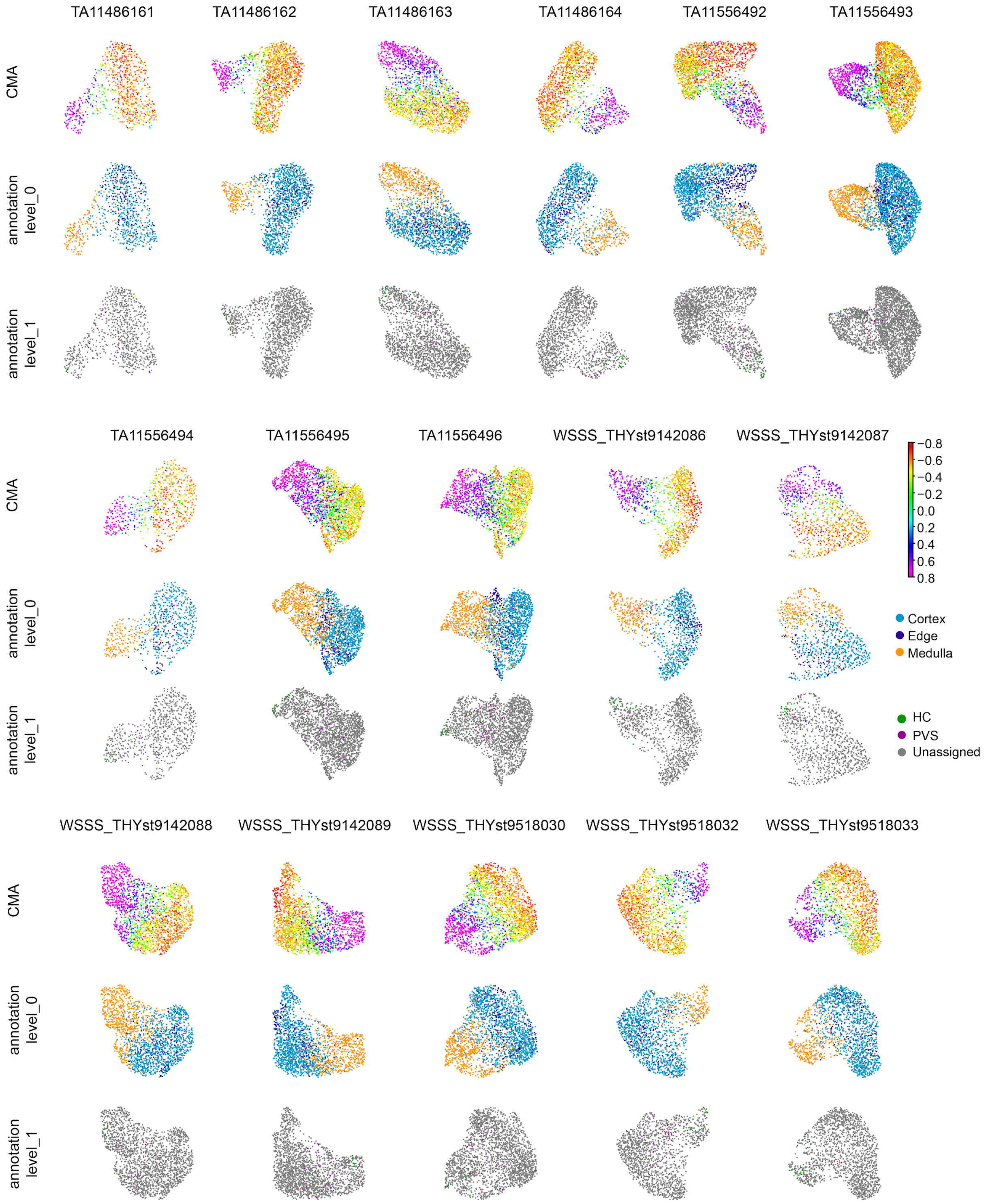

Supplementary Figure 9. UMAP plots for 16 individual paediatric Visium samples highlighting the major tissue annotations: CMA, annotation level 0, and annotation level 1.

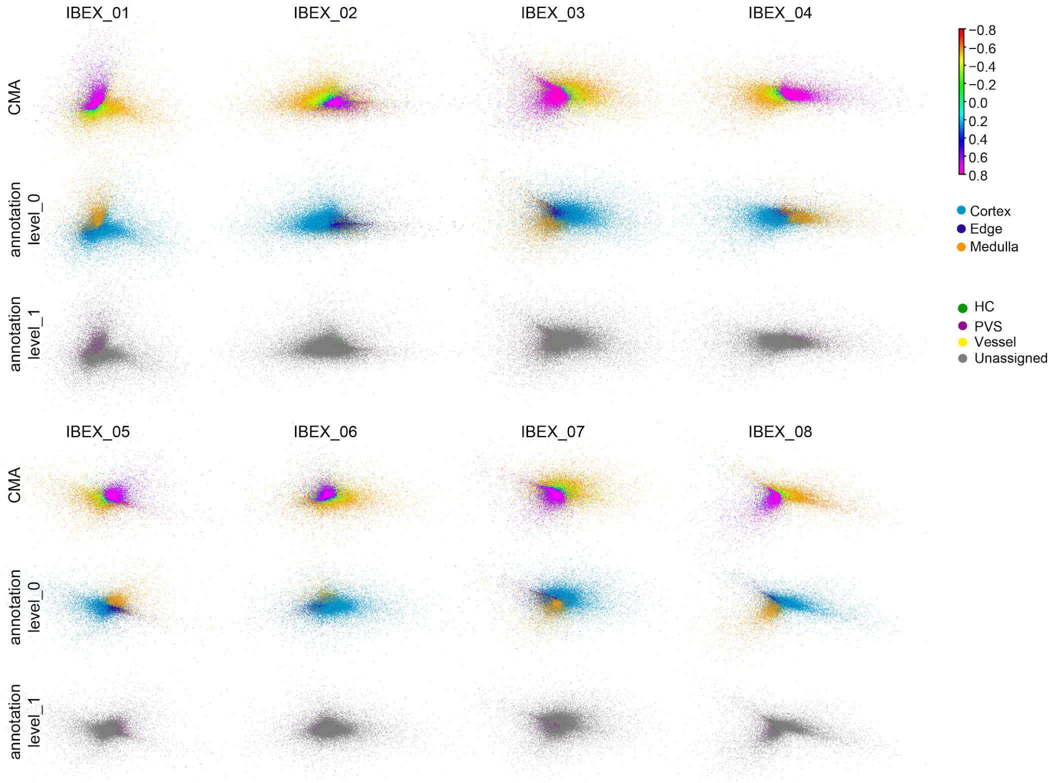

Supplementary Figure 10. PCA plots for 8 individual paediatric IBEX samples highlighting the major tissue annotations: CMA, annotation level 0, and annotation level 1.

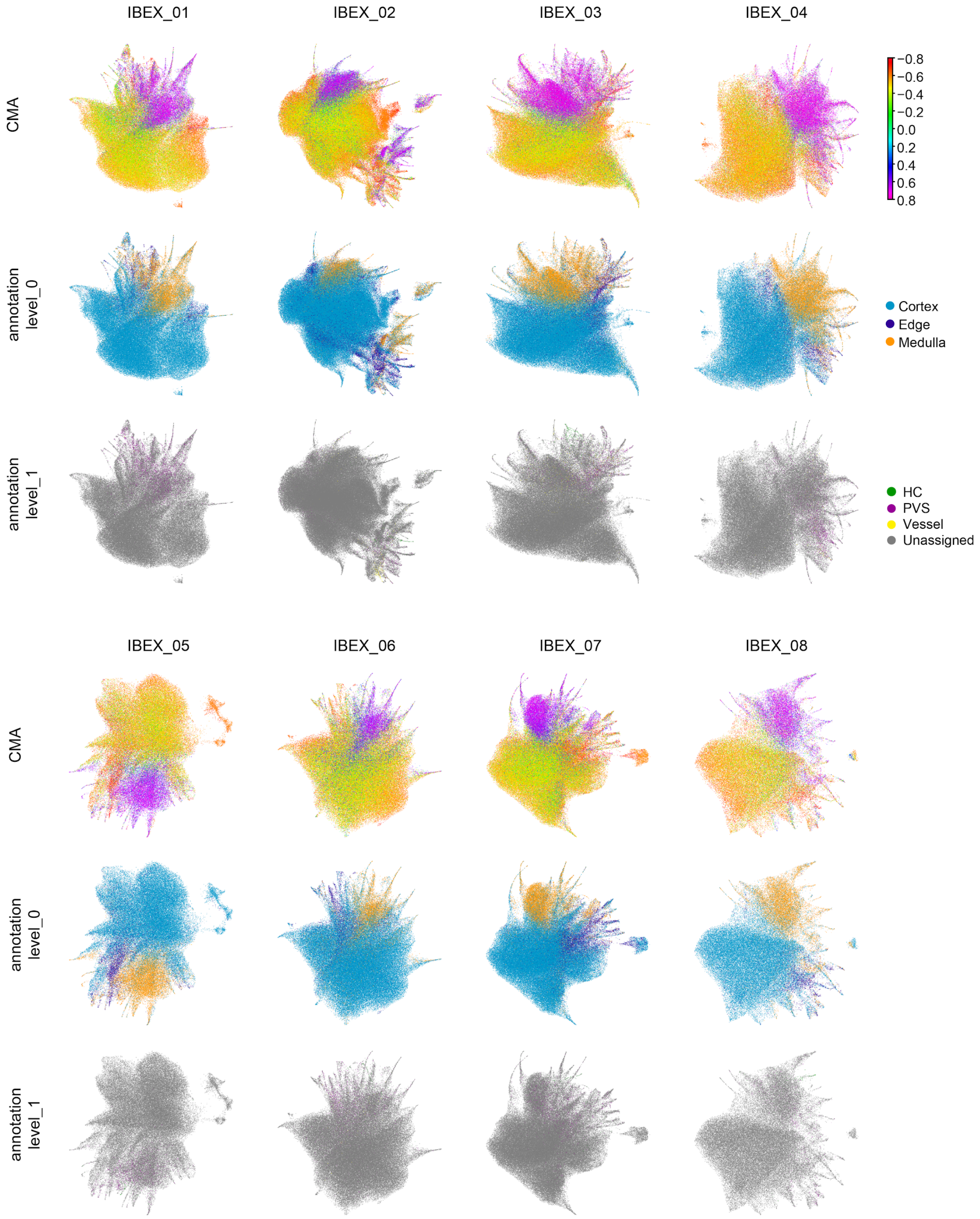

Supplementary Figure 11. UMAP plots for 8 individual paediatric IBEX samples highlighting the major tissue annotations: CMA, annotation level 0, and annotation level 1.

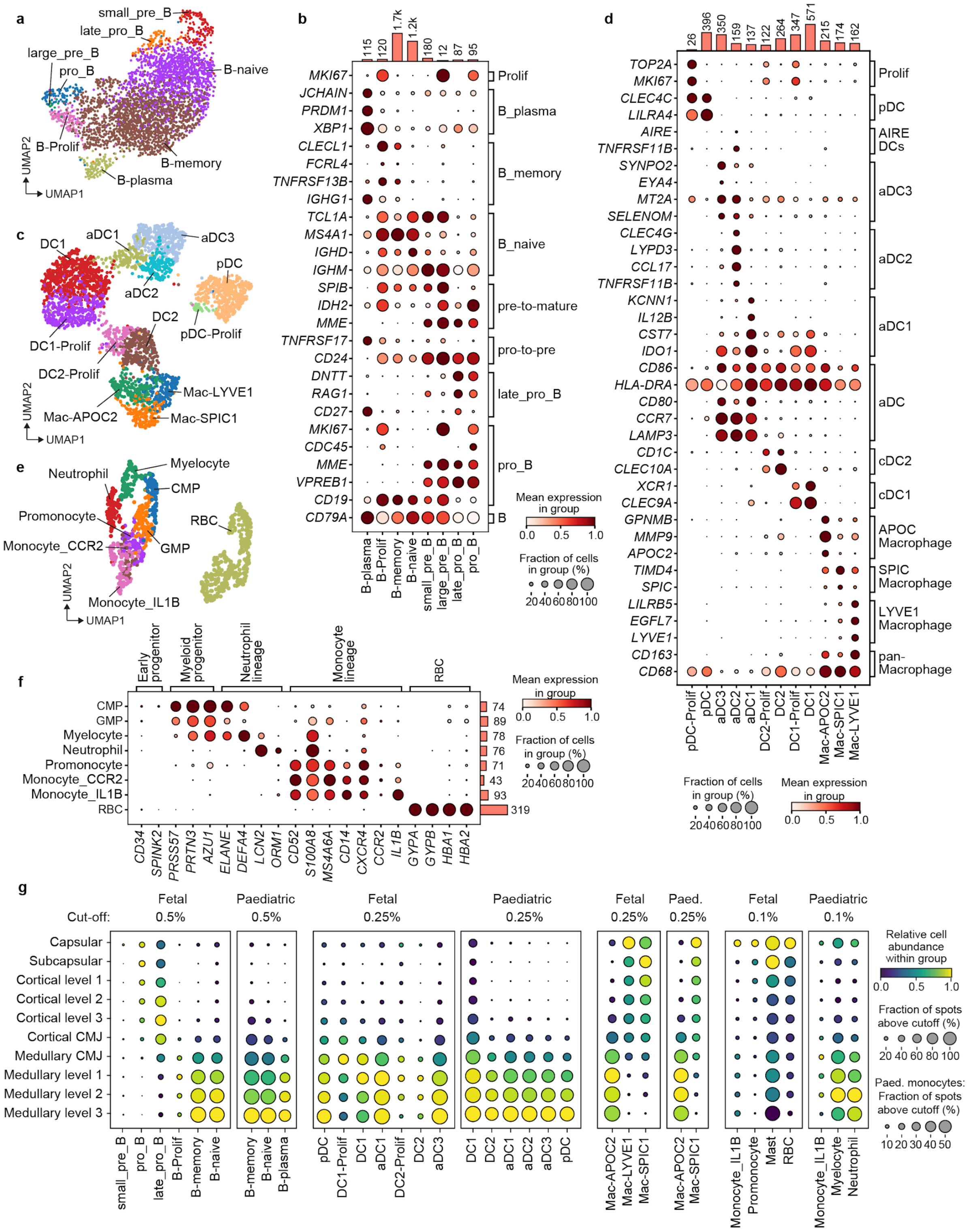
a
small_pre_B
late_pro_B
large_pre_B
pro_B
B-naive
B-Prolif
B-memory
B-plasma
UMAP2
UMAP1
b
115 120 1.7k 1.2k 180 12 87 95
MKI67
JCHAIN
PRDM1
XBP1
CLECL1
FCRL4
TNFRSF13B
IGHG1
TCL1A
MS4A1
IGHD
IGHM
SPIB
IDH2
MME
TNFRSF17
CD24
DNTT
RAG1
CD27
MKI67
CDC45
MME
VPREB1
CD19
CD79A
Prolif
B_plasma
B_memory
B_naive
pre-to-mature
pro-to-pre
late_pro_B
pro_B
B
B-plasma
B-Prolif
B-memory
B-naive
small_pre_B
large_pre_B
late_pro_B
pro_B
Mean expression in group
0.0 0.5 1.0
Fraction of cells in group (%)
20 40 60 80 100
c
DC1
aDC1
aDC3
pDC
aDC2
DC2
pDC-Prolif
DC1-Prolif
DC2-Prolif
Mac-LYVE1
Mac-APOC2
Mac-SPIC1
UMAP2
UMAP1
d
26 396 350 159 137 122 264 347 571 215 174 162
TOP2A
MKI67
CLEC4C
LILRA4
AIRE
TNFRSF11B
SYNPO2
EYA4
MT2A
SELENOM
CLEC4G
LYPD3
CCL17
TNFRSF11B
KCNN1
IL12B
CST7
IDO1
CD86
HLA-DRA
CD80
CCR7
LAMP3
CD1C
CLEC10A
XCR1
CLEC9A
GPNMB
MMP9
APOC2
TIMD4
SPIC
LILRB5
EGFL7
LYVE1
CD163
CD68
Prolif
pDC
AIRE DCs
aDC3
aDC2
aDC1
aDC
cDC2
cDC1
APOC Macrophage
SPIC Macrophage
LYVE1 Macrophage
pan-Macrophage
pDC-Prolif
pDC
aDC3
aDC2
aDC1
DC2-Prolif
DC2
DC1-Prolif
DC1
Mac-APOC2
Mac-SPIC1
Mac-LYVE1
Fraction of cells in group (%)
20 40 60 80 100
Mean expression in group
0.0 0.5 1.0
e
Neutrophil
Myelocyte
CMP
Promonocyte
RBC
Monocyte_CCR2
GMP
Monocyte_IL1B
UMAP2
UMAP1
f
Early progenitor
Myeloid progenitor
Neutrophil lineage
Monocyte lineage
RBC
CMP
GMP
Myelocyte
Neutrophil
Promonocyte
Monocyte_CCR2
Monocyte_IL1B
RBC
74 89 78 76 71 43 93 319
CD34
SPINK2
PRSS57
PRTN3
AZU1
ELANE
DEFA4
LCN2
ORM1
CD52
S100A8
MS4A6A
CD14
CXCR4
CCR2
IL1B
GYPA
GYPB
HBA1
HBA2
Mean expression in group
0.0 0.5 1.0
Fraction of cells in group (%)
20 40 60 80 100
g
Cut-off:
Fetal 0.5%
Paediatric 0.5%
Fetal 0.25%
Paediatric 0.25%
Fetal 0.25%
Paed. 0.25%
Fetal 0.1%
Paediatric 0.1%
Capsular
Subcapsular
Cortical level 1
Cortical level 2
Cortical level 3
Cortical CMJ
Medullary CMJ
Medullary level 1
Medullary level 2
Medullary level 3
small_pre_B
pro_B
late_pro_B
B-Prolif
B-memory
B-naive
B-memory
B-naive
B-plasma
pDC
DC1-Prolif
DC1
aDC1
DC2-Prolif
DC2
aDC3
DC1
DC2
aDC1
aDC2
aDC3
pDC
Mac-APOC2
Mac-LYVE1
Mac-SPIC1
Mac-APOC2
Mac-SPIC1
Monocyte_IL1B
Promonocyte
Mast
RBC
Monocyte_IL1B
Myelocyte
Neutrophil
Relative cell abundance within group
0.0 0.5 1.0
Fraction of spots above cutoff (%)
20 40 60 80 100
Paed. monocytes: Fraction of spots above cutoff (%)
10 20 30 40 50

Supplementary Figure 12. Fine-grained annotation and spatial mapping of hematopoietic cells in the fetal and paediatric human thymus. a. UMAP embedding of thymic B cell scRNA-seq data with annotations. **b.** Dot plot showing expression of marker genes in the annotated thymic B cell subsets in a. Total cell numbers per cell type are indicated by a bar graph. **c.** UMAP embedding of scRNA-seq data for thymic macrophages (“Mac”) and DCs with annotations. **d.** Dot plot showing expression of marker genes in the annotated DC and macrophage subsets in c. Total cell numbers per cell type are indicated by a bar graph. **e.** UMAP embedding of scRNA-seq data for additional thymic myeloid cells with annotations. CMP, common myeloid progenitor; GMP, granulocyte/monocyte progenitor; RBC, red blood cell. **f.** Dot plot showing expression of marker genes in the annotated thymic myeloid cell subsets in e. Total cell numbers per cell type are indicated by a bar graph. **g.** Predicted spatial mapping of the cells shown in a,c,e. after deconvolution of fetal and paediatric Visium data. Cut-off indicates the minimum proportion of the respective cell type in a Visium spot for the spot to be included. Dot size represents the proportion of spots meeting the cut-off and colour indicates the relative cell abundance. Note that not all cells in a,c,e were mapped to Visium (see **Supplementary Table 9**).

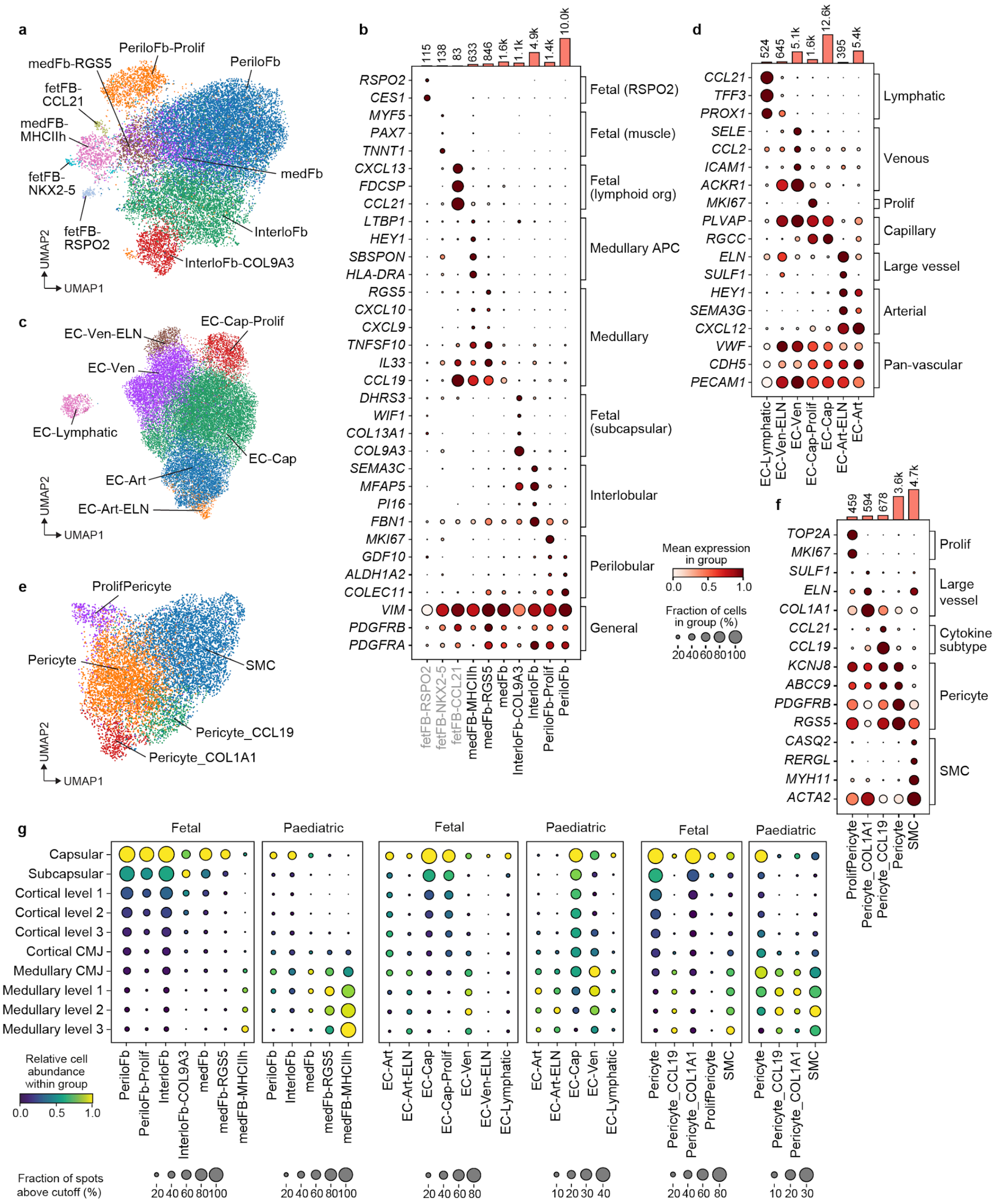

a
PerioFb-Prolif
medFb-RGS5
PerioFb
fetFB-CCL21
medFB-MHCIIh
medFb
fetFB-NKX2-5
fetFB-RSPO2
InterloFb
InterloFb-COL9A3
UMAP2
UMAP1
c
EC-Cap-Prolif
EC-Ven-ELN
EC-Ven
EC-Lymphatic
EC-Cap
EC-Art
EC-Art-ELN
e
ProlifPericyte
Pericyte
SMC
Pericyte_CCL19
Pericyte_COL1A1
b
RSPO2
CES1
MYF5
PAX7
TNNT1
CXCL13
FDCSP
CCL21
LTBP1
HEY1
SBSPON
HLA-DRA
RGS5
CXCL10
CXCL9
TNFSF10
IL33
CCL19
DHRS3
WIF1
COL13A1
COL9A3
SEMA3C
MFAP5
PI16
FBN1
MKI67
GDF10
ALDH1A2
COLEC11
VIM
PDGFRB
PDGFRA
115 138 83 633 846 1.6k 1.1k 4.9k 1.4k 10.0k
Fetal (RSPO2)
Fetal (muscle)
Fetal (lymphoid org)
Medullary APC
Medullary
Fetal (subcapsular)
Interlobular
Perilobular
General
fetFB-RSPO2
fetFB-NKX2-5
fetFB-CCL21
medFB-MHCIIh
medFb-RGS5
medFb
InterloFb-COL9A3
InterloFb
PerioFb-Prolif
PerioFb
Mean expression in group
0.0 0.5 1.0
Fraction of cells in group (%)
20 40 60 80 100
d
524 645 5.1k 1.6k 12.6k 395 5.4k
CCL21
TFF3
PROX1
SELE
CCL2
ICAM1
ACKR1
MKI67
PLVAP
RGCC
ELN
SULF1
HEY1
SEMA3G
CXCL12
VWF
CDH5
PECAM1
Lymphatic
Venous
Prolif
Capillary
Large vessel
Arterial
Pan-vascular
EC-Lymphatic
EC-Ven-ELN
EC-Ven
EC-Cap-Prolif
EC-Cap
EC-Art-ELN
EC-Art
f
459 594 678 3.6k 4.7k
TOP2A
MKI67
SULF1
ELN
COL1A1
CCL21
CCL19
KCNJ8
ABCC9
PDGFRB
RGS5
CASQ2
RERGL
MYH11
ACTA2
Prolif
Large vessel
Cytokine subtype
Pericyte
SMC
ProlifPericyte
Pericyte_COL1A1
Pericyte_CCL19
Pericyte
SMC
g
Fetal
Paediatric
Capsular
Subcapsular
Cortical level 1
Cortical level 2
Cortical level 3
Cortical CMJ
Medullary CMJ
Medullary level 1
Medullary level 2
Medullary level 3
PerioFb
PerioFb-Prolif
InterloFb
InterloFb-COL9A3
medFb
medFb-RGS5
medFB-MHCIIh
EC-Art
EC-Art-ELN
EC-Cap
EC-Cap-Prolif
EC-Ven
EC-Ven-ELN
EC-Lymphatic
Pericyte
Pericyte_CCL19
Pericyte_COL1A1
ProlifPericyte
SMC
Relative cell abundance within group
0.0 0.5 1.0
Fraction of spots above cutoff (%)
20 40 60 80 100
20 40 60 80
10 20 30 40
10 20 30

Supplementary Figure 13. Annotation and spatial mapping of thymic fibroblasts and vascular cells. a. UMAP embedding of thymic fibroblast (Fb) scRNA-seq data with annotations. medFB, medullary fibroblast; fetFB, fetal fibroblast. **b.** Dot plot showing expression of marker genes in the annotated thymic fibroblast subsets in a. Total cell numbers per cell type are indicated by a bar graph. **c.** UMAP embedding of thymic endothelial cell (EC) scRNA-seq data with annotations. Ven, venous; Art, arterial; Cap, capillary. **d.** Dot plot showing expression of marker genes in the annotated thymic endothelial cell subsets in c. Total cell numbers per cell type are indicated by a bar graph. **e.** UMAP embedding of thymic smooth muscle cell (SMC) scRNA-seq data with annotations. **f.** Dot plot showing expression of marker genes in the annotated thymic smooth muscle cell subsets in e. Total cell numbers per cell type are indicated by a bar graph. **g.** Predicted spatial mapping of thymic fibroblast, endothelial and smooth muscle cells after deconvolution of fetal and paediatric Visium data. A cut-off of 0.15 was used and indicates the minimum proportion of the respective cell type in a Visium spot for the spot to be included. Dot size represents the proportion of spots meeting the cut-off and colour indicates the relative cell abundance.

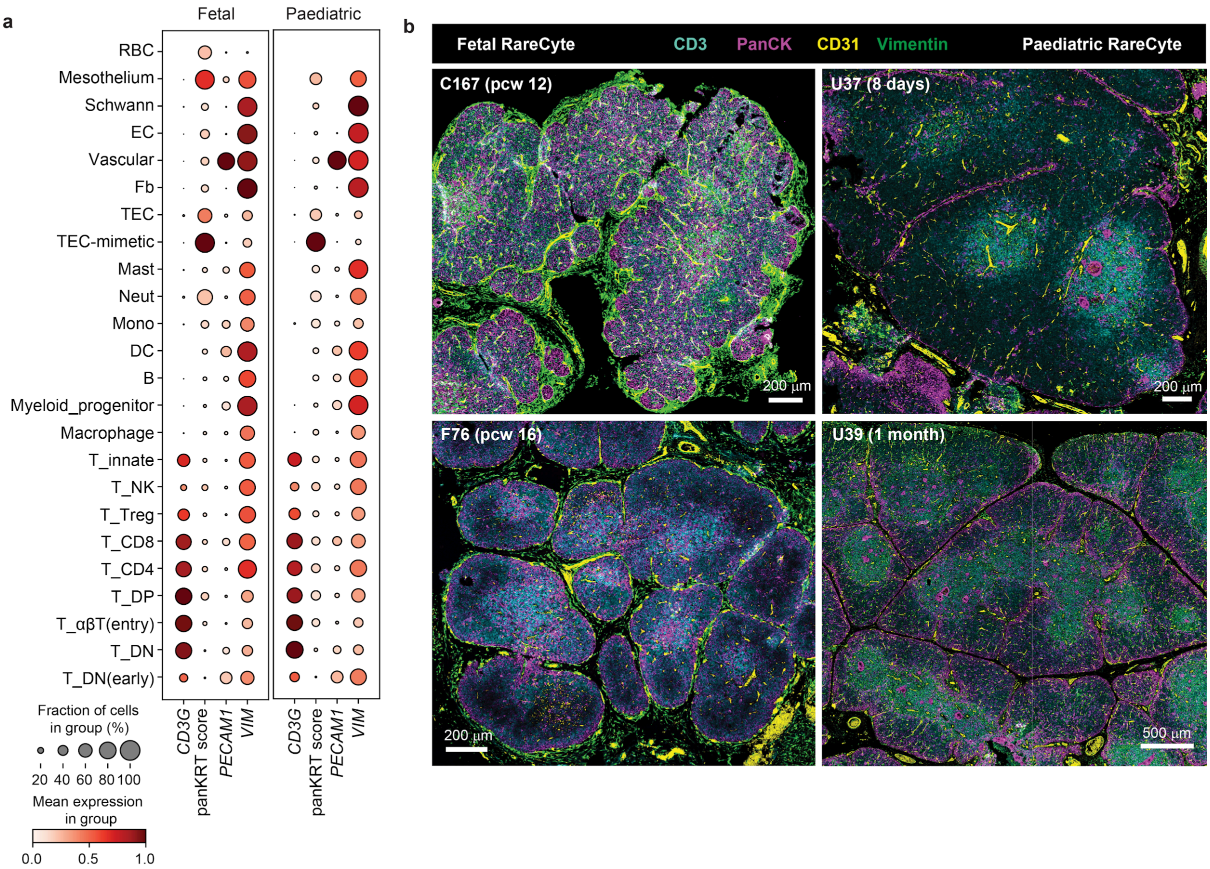

Supplementary Figure 14. Capsular and CMJ regions in fetal and paediatric thymus. a. Expression of *CD3G*, *PECAM1* (encoding CD31), *VIM* genes in various cell lineages in the scRNA-seq dataset. Keratin gene set scoring was performed to derive a pan-KRT score, which would correspond to Pan-Cytokeratin protein staining. RBC, red blood cell; EC, endothelial cell; Fb, fibroblast. **b.** Expression of corresponding proteins as evaluated using 4-plex RareCyte staining on fetal (p.c.w. 12 and p.c.w. 16) and paediatric (8 days and 1 month old) thymus sections. Images are representative of 6 fetal and 3 paediatric replicates.

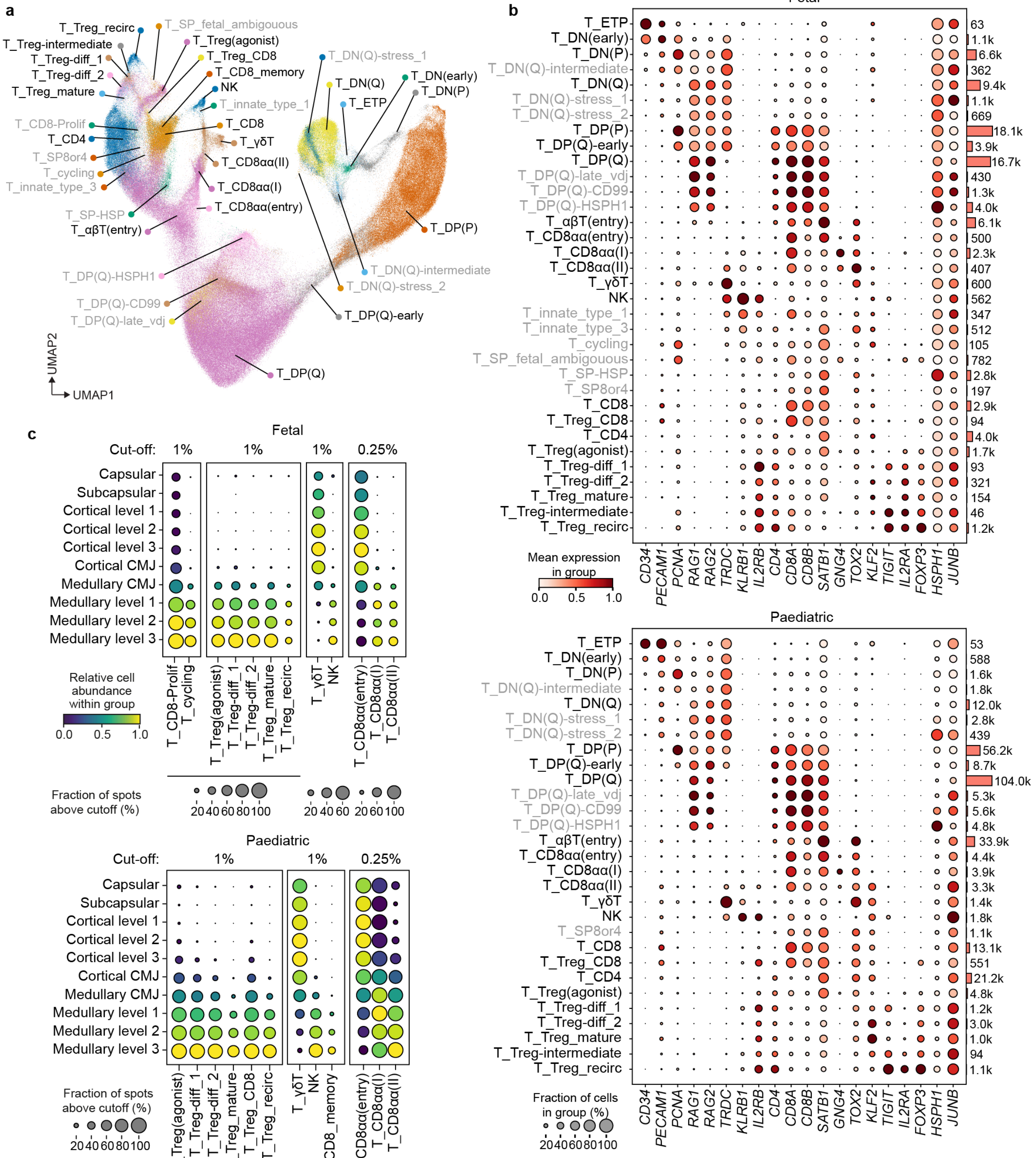
a
T_Treg_recirc
T_SP_fetal_ambigouous
T_Treg-intermediate
T_Treg(agonist)
T_Treg-diff_1
T_Treg_CD8
T_Treg-diff_2
T_CD8_memory
T_Treg_mature
NK
T_innate_type_1
T_CD8-Prolif
T_CD8
T_CD4
T_γδT
T_SP8or4
T_CD8αα(II)
T_cycling
T_innate_type_3
T_CD8αα(I)
T_SP-HSP
T_CD8αα(entry)
T_αβT(entry)
T_DN(Q)-stress_1
T_DN(Q)
T_DN(early)
T_DN(P)
T_ETP
T_DP(P)
T_DN(Q)-intermediate
T_DN(Q)-stress_2
T_DP(Q)-HSPH1
T_DP(Q)-CD99
T_DP(Q)-late_vdj
T_DP(Q)-early
T_DP(Q)
UMAP2
UMAP1
b
Fetal
T_ETP 63
T_DN(early) 1.1k
T_DN(P) 6.6k
T_DN(Q)-intermediate 362
T_DN(Q) 9.4k
T_DN(Q)-stress_1 1.1k
T_DN(Q)-stress_2 669
T_DP(P) 18.1k
T_DP(Q)-early 3.9k
T_DP(Q) 16.7k
T_DP(Q)-late_vdj 430
T_DP(Q)-CD99 1.3k
T_DP(Q)-HSPH1 4.0k
T_αβT(entry) 6.1k
T_CD8αα(entry) 500
T_CD8αα(I) 2.3k
T_CD8αα(II) 407
T_γδT 600
NK 562
T_innate_type_1 347
T_innate_type_3 512
T_cycling 105
T_SP_fetal_ambigouous 782
T_SP-HSP 2.8k
T_SP8or4 197
T_CD8 2.9k
T_Treg_CD8 94
T_CD4 4.0k
T_Treg(agonist) 1.7k
T_Treg-diff_1 93
T_Treg-diff_2 321
T_Treg_mature 154
T_Treg-intermediate 46
T_Treg_recirc 1.2k
CD34 PECAM1 PCNA RAG1 RAG2 TRDC KLRB1 IL2RB CD4 CD8A CD8B SATB1 GNG4 TOX2 KLF2 TIGIT IL2RA FOXP3 HSPH1 JUNB
Mean expression in group
0.0 0.5 1.0
Paediatric
T_ETP 53
T_DN(early) 588
T_DN(P) 1.6k
T_DN(Q)-intermediate 1.8k
T_DN(Q) 12.0k
T_DN(Q)-stress_1 2.8k
T_DN(Q)-stress_2 439
T_DP(P) 56.2k
T_DP(Q)-early 8.7k
T_DP(Q) 104.0k
T_DP(Q)-late_vdj 5.3k
T_DP(Q)-CD99 5.6k
T_DP(Q)-HSPH1 4.8k
T_αβT(entry) 33.9k
T_CD8αα(entry) 4.4k
T_CD8αα(I) 3.9k
T_CD8αα(II) 3.3k
T_γδT 1.4k
NK 1.8k
T_SP8or4 1.1k
T_CD8 13.1k
T_Treg_CD8 551
T_CD4 21.2k
T_Treg(agonist) 4.8k
T_Treg-diff_1 1.2k
T_Treg-diff_2 3.0k
T_Treg_mature 1.0k
T_Treg-intermediate 94
T_Treg_recirc 1.1k
CD34 PECAM1 PCNA RAG1 RAG2 TRDC KLRB1 IL2RB CD4 CD8A CD8B SATB1 GNG4 TOX2 KLF2 TIGIT IL2RA FOXP3 HSPH1 JUNB
Fraction of cells in group (%)
20 40 60 80 100
c
Fetal
Cut-off: 1% 1% 1% 0.25%
Capsular
Subcapsular
Cortical level 1
Cortical level 2
Cortical level 3
Cortical CMJ
Medullary CMJ
Medullary level 1
Medullary level 2
Medullary level 3
T_CD8-Prolif
T_cycling
T_Treg(agonist)
T_Treg-diff_1
T_Treg-diff_2
T_Treg_mature
T_Treg_recirc
T_γδT
NK
T_CD8αα(entry)
T_CD8αα(I)
T_CD8αα(II)
Relative cell abundance within group
0.0 0.5 1.0
Fraction of spots above cutoff (%)
20 40 60 80 100
20 40 60
20 60 100
Paediatric
Cut-off: 1% 1% 0.25%
Capsular
Subcapsular
Cortical level 1
Cortical level 2
Cortical level 3
Cortical CMJ
Medullary CMJ
Medullary level 1
Medullary level 2
Medullary level 3
T_Treg(agonist)
T_Treg-diff_1
T_Treg-diff_2
T_Treg_mature
T_Treg_CD8
T_Treg_recirc
T_γδT
NK
T_CD8_memory
T_CD8αα(entry)
T_CD8αα(I)
T_CD8αα(II)
Fraction of spots above cutoff (%)
20 40 60 80 100

Supplementary Figure 15. Full T lineage annotations in scRNA-seq data and spatial mapping via deconvolution of Visium data. a. UMAP embedding of integrated fetal and paediatric scRNA-seq data spanning the entire T lineage with the most detailed annotations of differentiation stages. Grey cell type labels correspond to cells which we could not confidently assign to a unique known cell type but can be explored in the provided AnnData object (“cell_type_level_4_explore”). recirc: recirculating; late-vdj: late V(D)J recombination pseudotime according to Dandelion (Ref. 47). **b.** Dot plot showing expression of marker genes across the entire T lineage for fetal (top) and paediatric data (bottom). Bar graph indicates total cell numbers per cell type. Grey cell type labels indicate cell subsets which we could not confidently assign to a unique known cell type based on their gene expression profiles. These can be explored in the provided AnnData object (“cell_type_level_4_explore”). **c.** Spatial mapping of T lineage cells through calculation of the CMA for Visium data and deconvolution based on the scRNA-seq reference shown in a. Cell subsets shown in Figure 3 are not included here. Cut-off indicates the minimum proportion of the respective cell type in a Visium spot for the spot to be included. Dot size represents the proportion of spots meeting the cut-off and colour indicates the relative cell abundance. Note that not all cells in “cell_type_level_4_explore” were mapped to Visium (see **Supplementary Table 9**).

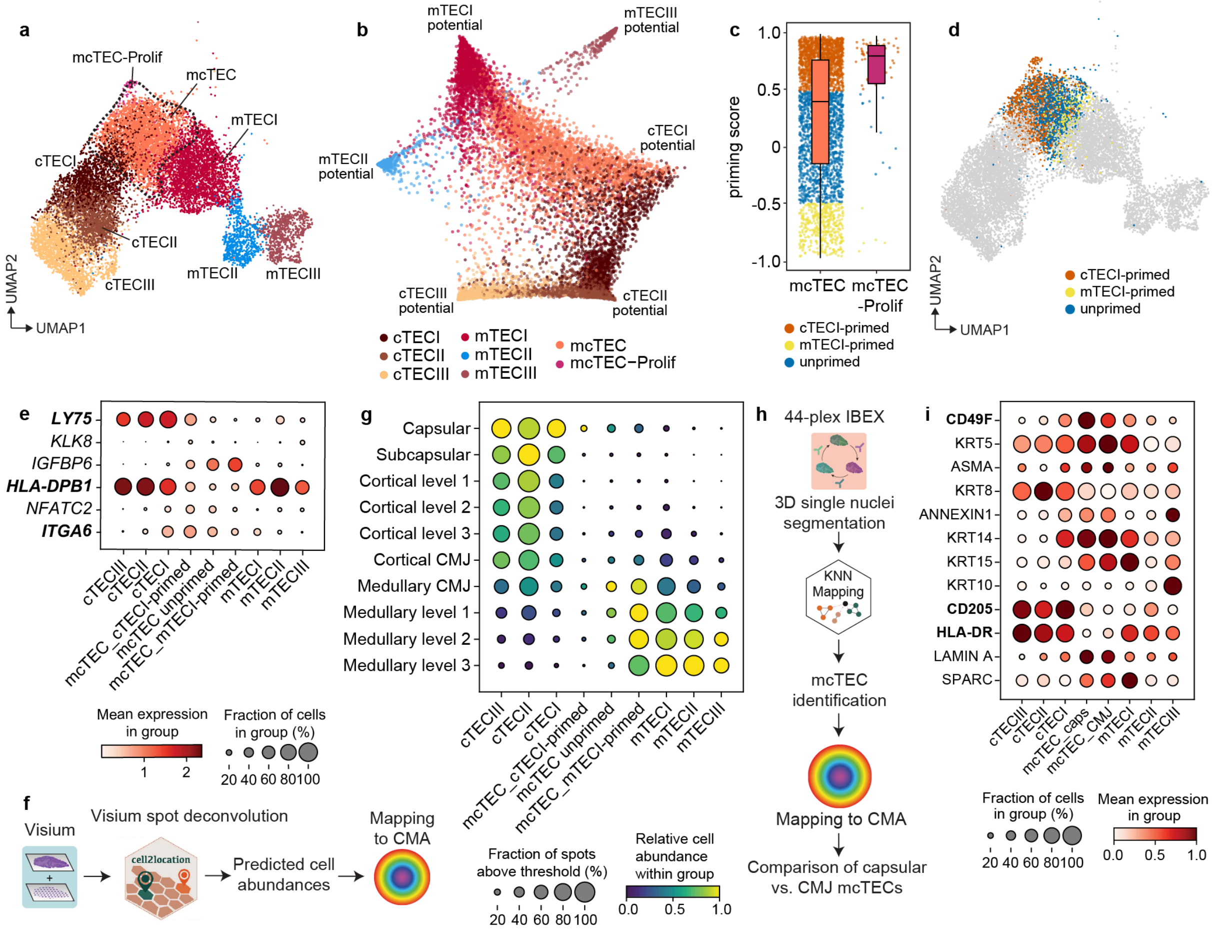

Supplementary Figure 16. Exploring the fate probabilities of paediatric mcTECs. a. UMAP embedding of scRNA-seq data from paediatric TECs coloured by annotation. **b.** STEMNET map depicting the putative potential of major TEC subtypes to differentiate into cTECI/II/III or mTECI/II/III. Cells are coloured by annotations. The six corners of the hexagonal simplex represent the highest probability for the six mature TEC fates. **c.** cTECI priming score for mcTEC and mcTEC-prolif subtypes. Cells were considered cTECI-primed or mTECI-primed if their priming score was above 0.5 or below -0.5, respectively. The number of mcTECs with high mTECII/III or cTECII/III potentials was negligible and therefore not included in this analysis. Box plot shows median priming score, hinges correspond to first and third quartile, whiskers indicate hinge + 1.5 times the interquartile range. **d.** Same UMAP embedding as in a. with mcTEC and mcTEC-Prolif coloured by their mTECI- vs. cTECI-priming as determined in c. **e.** Dot plot illustrating the most distinct genes between mTECI- vs cTECI-primed mcTECs. **f.** Workflow used to determine the spatial distribution of major TEC subtypes and primed/unprimed mcTECs. **g.** CMA mapping of major TEC subtypes and primed/unprimed mcTECs in the paediatric thymus. Dot size represents the proportion of spots with cell type proportion above the cut-off and colour indicates the relative cell abundance. **h.** Schematic depiction of the workflow used to determine capsular vs. CMJ localisation of mcTECs in paediatric IBEX. **i.** Dot plot showing expression of the most distinct protein markers between capsular and CMJ-associated mcTECs in IBEX. Proteins for which the corresponding gene expression in the scRNA-seq data is shown in e. are highlighted in bold.

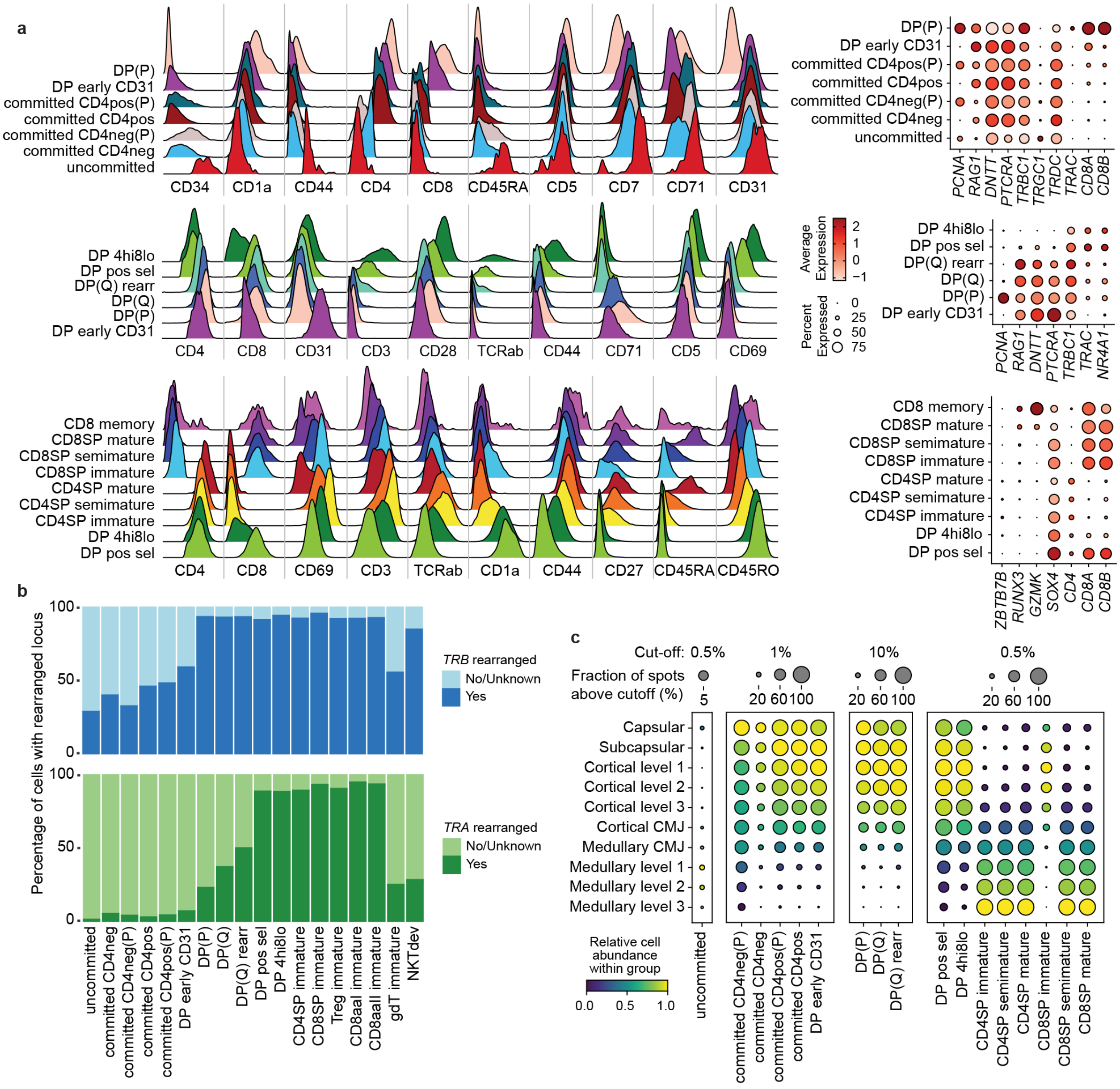

Supplementary Figure 17. Surface protein-guided annotations and spatial mapping of maturation stages of conventional T lineage cells in the paediatric thymus. a. Expression of selected markers relevant for the distinction and annotation of differentiation stages of immature, DP, and SP thymocytes. Density histograms indicate surface protein levels after denoising and normalisation (left). Dot plots show scaled normalised pseudobulk RNA levels for selected relevant genes (right). **b.** Bar graph illustrating the proportion of cells with rearranged *TRB* and *TRA* locus at selected maturation stages based on TCR-seq data analysis with Dandelion (Ref. 47). **c.** CMA mapping of the annotated stages of conventional T lineage development through deconvolution of paediatric Visium sections. Selected stages were plotted separately with adjusted cut-off to aid visualisation of smaller subsets. Cut-off indicates the minimum proportion of the respective cell type in a Visium spot for the spot to be included. Dot size represents the proportion of spots meeting the cut-off and colour indicates the relative cell abundance. DP, double positive; SP, single positive; pos_sel, positive-selected; (P), proliferating; (Q): quiescent; rearr, TCR-rearranging.

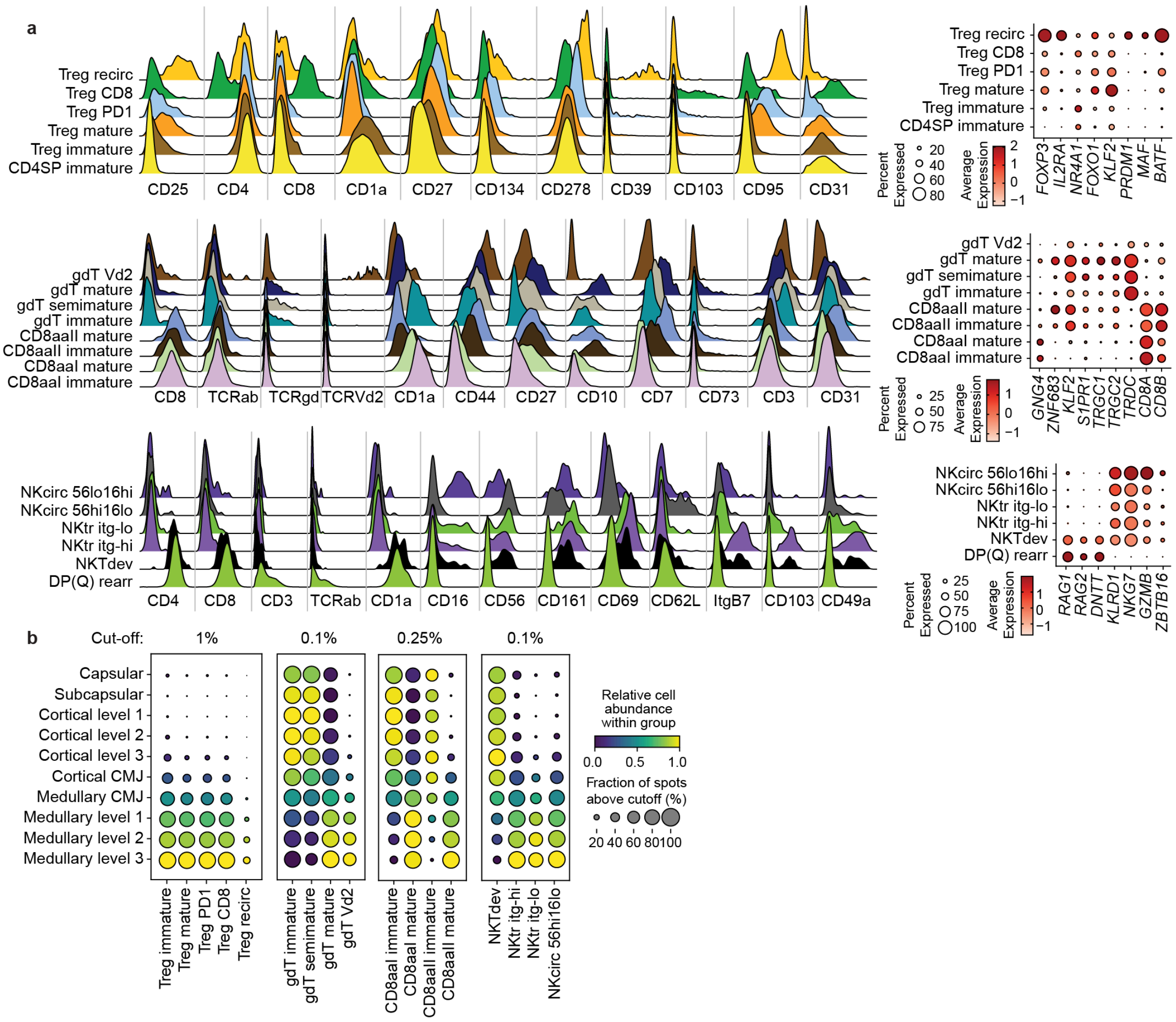

Supplementary Figure 18. Surface protein-guided annotations and spatial mapping of unconventional T and NK cells. a. Expression of selected markers relevant for the distinction and annotation of subtypes and differentiation stages of regulatory T cells, CD8αα T cells, γδ T cells, and NK(T) cells. Density histograms indicate surface protein levels after denoising and normalisation (left). Dot plots show scaled normalised pseudobulk RNA levels for selected relevant genes (right). **b.** CMA mapping of the annotated stages of conventional T lineage development through deconvolution of paediatric Visium sections. Subtypes were plotted separately with adjusted cut-off to aid visualisation of rare subsets. Cut-off indicates the minimum proportion of the respective cell type in a Visium spot for the spot to be included. Dot size represents the proportion of spots meeting the cut-off and colour indicates the relative cell abundance. Recirc, recirculating; tr, tissue resident; circ, circulating; itg, integrin.

a

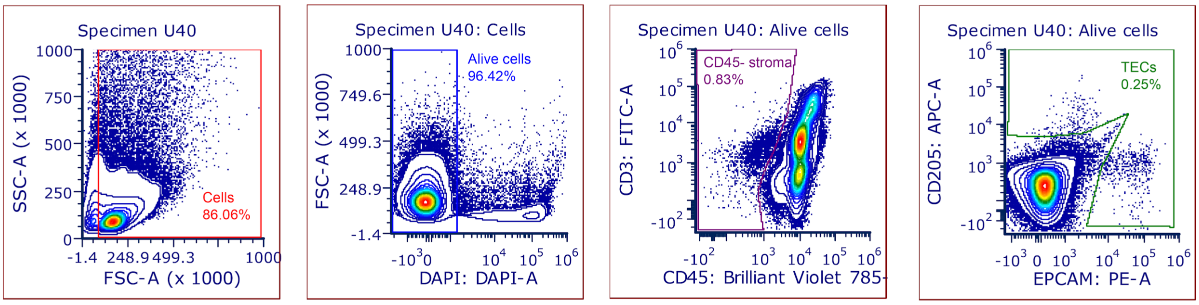

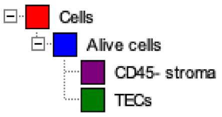

Gate	# of Events	X Geometric Mean	Y Geometric Mean	% of all cells
No Gate	304395	n/a	118683.54	100.00
Cells	261969	176065.37	112319.54	86.06
Alive cells	252590	178393.42	109786.99	82.98
CD45- stroma	2109	224202.52	359447.29	0.69
TECs	629	304347.30	410541.04	0.21

b

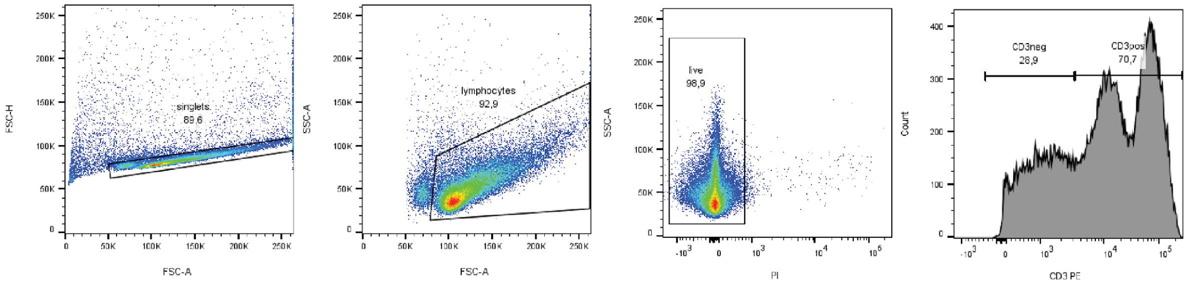

Supplementary Figure 19. Gating strategy for FACS sorting. a. Sorting scheme for stroma enrichment experiments. Cells were stained with a mix of anti-EPCAM PE, anti-CD205 APC, anti-CD45 BV785, anti-CD3 FITC and DAPI was used as a dead cell marker. No multiplet filtering was done to allow capture of large cTECs. First, $CD45^-$ cells were sorted to obtain total stroma. Next, a gate including either $CD205^+EPCAM^-$ or $CD205^-EPCAM^+$ cells was used to sort total thymic epithelial cells. $CD205^+EPCAM^+$ cells were not included since these were suspected to be autofluorescent cells or doublets. Plots are representative for all samples profiled in this study (see Figure 1c, Extended Data Figure 1a and Supplementary Table 1). **b.** Sorting scheme for CITE-seq experiments. Cells were stained with anti-CD3 PE and propidium iodide (PI) was used as a dead cell marker. For each sample debris, doublets and PI^+ cells were gated out and $CD3^+$ and $CD3^-$ cells were sorted and collected separately. Plot is representative for all CITE-seq samples profiled in this study (see Figure 1c, Extended Data Figure 1a and Supplementary Table 1).
