# Supplementary material for: A spatial human thymus cell atlas mapped to a continuous tissue axis
Source: Nature. 2024 Nov 20;635(8039):708–18. doi: 10.1038/s41586-024-07944-6 (PMC11578893; doi:10.1038/s41586-024-07944-6)
Supplement: Supplementary file 2 — Reporting Summary [file 41586_2024_7944_MOESM2_ESM.pdf]

nature portfolio

Corresponding author(s): Ronald Germain, Andrea J Radtke, John Marioni, Tom Taghon and Sarah Teichmann

Last updated by author(s): 11/07/2024

Reporting Summary

Nature Portfolio wishes to improve the reproducibility of the work that we publish. This form provides structure for consistency and transparency in reporting. For further information on Nature Portfolio policies, see our Editorial Policies and the Editorial Policy Checklist.

Statistics

For all statistical analyses, confirm that the following items are present in the figure legend, table legend, main text, or Methods section.

n/a	Confirmed	
☐	☒	The exact sample size (*n*) for each experimental group/condition, given as a discrete number and unit of measurement
☐	☒	A statement on whether measurements were taken from distinct samples or whether the same sample was measured repeatedly
☐	☒	The statistical test(s) used AND whether they are one- or two-sided<br>*Only common tests should be described solely by name; describe more complex techniques in the Methods section.*
☐	☒	A description of all covariates tested
☐	☒	A description of any assumptions or corrections, such as tests of normality and adjustment for multiple comparisons
☐	☒	A full description of the statistical parameters including central tendency (e.g. means) or other basic estimates (e.g. regression coefficient) AND variation (e.g. standard deviation) or associated estimates of uncertainty (e.g. confidence intervals)
☐	☒	For null hypothesis testing, the test statistic (e.g. *F*, *t*, *r*) with confidence intervals, effect sizes, degrees of freedom and *P* value noted<br>*Give P values as exact values whenever suitable.*
☒	☐	For Bayesian analysis, information on the choice of priors and Markov chain Monte Carlo settings
☒	☐	For hierarchical and complex designs, identification of the appropriate level for tests and full reporting of outcomes
☒	☐	Estimates of effect sizes (e.g. Cohen's *d*, Pearson's *r*), indicating how they were calculated

Our web collection on statistics for biologists contains articles on many of the points above.

Software and code

Policy information about availability of computer code

Data collection	The following packages were used to collect the publicly available data: srapath v2.11.0, bamtofastq v1.3.2, entrez-direct v15.6, fastq-dump v2.11.0, samtools v1.12 and iRODs v4.2.7.
Data analysis	All code scripts and notebooks used in the study are publicly available here: https://github.com/Teichlab/thymus_spatial_atlas. Code for the TissueTag package is available in the following GitHub repository: https://github.com/Teichlab/TissueTag.<br>We also list the major libraries used below:<br>- Data mapping and pre-processing: STARsolo with STAR v2.7.9a, Cell Ranger v6.1.1 and v7.0.0 ; soup removal: CellBender v0.1.0;<br>- Python libraries:<br>general: pandas v2.2.2, numpy v1.26.4, scipy v1.13.0, scikit-learn v1.4.2, seaborn v0.13.2, matplotlib v3.8.4; single-cell processing: scanpy v1.9.1 with anndata v0.10.7, doublet removal: scrublet v0.2.3, automatic cell annotation: celltypist v1.6.2, data integration and embedding: scvi-tools 0.19.0; trajectory inference: scFates v1.0.7 and Palantir v1.3.3; knn mapping: https://github.com/Teichlab/iss_patcher/tree/main.<br>- R libraries:<br>general: Matrix (v1.6-4), matrixStats (v1.2.0), dplyr (v1.1.4), tidyr (v1.3.1), reshape2 (v1.4.4), BiocNeighbours (v1.20.2), BiocParallel (v1.36.0), stringr (1.5.1), reticulate (v1.35.0), and sceasy (v0.0.7); visualisation: ggplot2 (v3.5.0), ggrastr (v1.0.2), ggridges (v0.5.6) and RColorBrewer (v1.1-3); single-cell processing: Seurat (v4.3.0), SeuratObject (v4.1.4), SeuratDisk (v0.0.0.9021), SingleCellExperiment (v1.24.0), sceasy (v0.0.7); CITEseq denoising: dsb v1.0.3; batch correction: Batchelor v1.18.1; marker identification: singleCellHaystack v1.0.0, trajectory inference: Slingshot v2.6.0; fate mapping: STEMNET v0.1.<br>- TCR data processing: https://github.com/zktuong/dandelion v0.3.1.<br>- Spatial data processing: SpaceRanger v1.2.2 and v1.3.0, cell2location v0.1.3.<br>- Image processing: https://github.com/Teichlab/TissueTag or tissue-tag 0.1.1 uses bokeh v3.4.1, scikit-image v0.22.0, cellpose v2.1.1.IBEX

data processing: LAS X Navigator v3.5.7.23225, Fiji 1.54j, IMARIS. RareCyte: Artemis v4.
- FACS data acquisition and analysis: FACSDiva v8.0.2, FlowJo v10.8.2 and FCS Express v7.18.0025.

For manuscripts utilizing custom algorithms or software that are central to the research but not yet described in published literature, software must be made available to editors and reviewers. We strongly encourage code deposition in a community repository (e.g. GitHub). See the Nature Portfolio guidelines for submitting code & software for further information.

Data

Policy information about availability of data

All manuscripts must include a data availability statement. This statement should provide the following information, where applicable:

- Accession codes, unique identifiers, or web links for publicly available datasets
- A description of any restrictions on data availability
- For clinical datasets or third party data, please ensure that the statement adheres to our policy

The annotated fetal and paediatric integrated scRNA-seq atlas and Visium objects for this study can be explored online https://cellxgene.cziscience.com/collections/fc19ae6c-d7c1-4dce-b703-62c5d52061b4.
Paediatric CITE-seq data can be visualised and explored using acustom ShinyApp (https://ccgg.ugent.be/shiny/htsa_thymocyte_citeseq/). Sequencing data for the newly generated libraries for scRNA-seq and Visium data were uploaded to ENA under accession code PRJEB77091. Several samples were obtained under consent agreements that require data release with managed access, which is why these were deposited at EGA under accession code EGAD00001015384.
Imaging data for Visium samples were deposited at BioImage Archive under accession number S-BIAD1257. CITE-seq data were uploaded to the GEO under accession number GSE271304. Imaging data generated for this study, including IBEX, RNAscope and RareCyte data, were deposited at the BioImage Archive under the accession number S-BIAD1257. Publicly available datasets were downloaded from the following sources: ref. 3 (ArrayExpress: E-MTAB-8581; GEO: GSE206710); ref. 15 (GEO: GSE159745); ref. 7 (GEO: GSE147520); and ref. 16 (ArrayExpress:E-MTAB-11341). All accession codes are also listed in Supplementary Tables 1 and 2. Source data are provided with this paper.

Research involving human participants, their data, or biological material

Policy information about studies with human participants or human data. See also policy information about sex, gender (identity/presentation), and sexual orientation and race, ethnicity and racism.

Reporting on sex and gender	1) scRNAseq: 6 male and 7 female fetal samples and 10 male and 8 female paediatric samples were used to generate the reference thymus scRNA-seq atlas.<br>2) Visium spatial transcriptomics: samples from 7 fetal donors were used (sex wasn't recorded) as well as 2 male and 4 female donors.<br>3) IBEX: samples from 4 male and 4 female patients were used.<br>Sex comparison wasn't performed.
Reporting on race, ethnicity, or other socially relevant groupings	Data on race, ethnicity and socioeconomics status wasn't collected.
Population characteristics	For the samples used for to put together reference single-cell and Visium spatial transcriptomics atlas age was recorded and used as covariate during the sample integrations.<br>1) scRNAseq: fetal donors ranged in age from 11 pcw to 21 pcw, while paediatric patients ranged from 6 days until 2.5 yrs.<br>2) Visium: fetal donors ranged in age from 12 until 18 pcw, while paediatric patients ranged in age from 3 months to 2.5 yrs.<br>3) IBEX: patients ranged in age from 7 days to 1 year.
Recruitment	Paediatric thymi samples were obtained from the patients undergoing corrective cardiac surgeries. Fetal samples were donated voluntarily by women who have had a termination of pregnancy from clinics collaborating with the Human Developmental Biology Resource, UK (https://www.hdbr.org/).
Ethics oversight	Wellcome Sanger Institute, UK: Paediatric thymi samples were provided by Newcastle University collected under REC approved study 18/EM/0314 and Great Ormond Street Hospital under REC approved study 07/Q0508/43. The human embryonic and fetal material was provided by the joint MRC / Wellcome Trust (Grant # MR/006237/1) Human Developmental Biology Resource (http://www.hdbr.org).<br><br>Ghent University, Belgium: Paediatric thymi samples were obtained according to and used with the approval of the Medical Ethical Commission of Ghent University Hospital, Belgium (EC/2019-0826) through the hematopoietic cell biobank (EC-Bio/1-2018).<br><br>NIH, US: Paediatric thymi samples processed at the NIH were obtained (under NIAID MTA 2016-250) from the pathology department of the Children's National Medical Center in Washington, DC, following cardiothoracic surgery from children with congenital heart disease. Use of these thymus samples for this study was determined to be exempt from review by the NIH Institutional Review Board in accordance with the guidelines issued by the Office of Human Research Protections.<br><br>Written informed consent from the donors or their families was obtained for the samples obtained at all the sites.

Note that full information on the approval of the study protocol must also be provided in the manuscript.

Field-specific reporting

Please select the one below that is the best fit for your research. If you are not sure, read the appropriate sections before making your selection.

☒ Life sciences ☐ Behavioural & social sciences ☐ Ecological, evolutionary & environmental sciences

For a reference copy of the document with all sections, see nature.com/documents/nr-reporting-summary-flat.pdf

Life sciences study design

All studies must disclose on these points even when the disclosure is negative.

Sample size	No sample size calculation was performed, sample size was determined by the number of integrated datasets and time availability to collect new ones.<br>scRNA-seq: 13 fetal donors and 18 paediatric donors;<br>Visium: 7 fetal donors and 6 paediatric donors;<br>IBEX: 8 paediatric patients.
Data exclusions	No samples were excluded from the study.
Replication	Validation using alternative approaches was performed to confirm the reproducibility of the findings, including using RNAscope (n=4 fetal donors) and RareCyte experiments (6 fetal donors and 3 paediatric).
Randomization	Randomization wasn't performed as it is not applicable to this project. Samples were allocated into fetal and pediatric age groups based on their origin before or after birth.
Blinding	Blinding for analysis of spatial data wasn't possible due to large size and morphological differences between fetal and paediatric thymus.

Reporting for specific materials, systems and methods

We require information from authors about some types of materials, experimental systems and methods used in many studies. Here, indicate whether each material, system or method listed is relevant to your study. If you are not sure if a list item applies to your research, read the appropriate section before selecting a response.

Materials & experimental systems

n/a	Involved in the study	
☐	☒	Antibodies
☒	☐	Eukaryotic cell lines
☒	☐	Palaeontology and archaeology
☒	☐	Animals and other organisms
☒	☐	Clinical data
☒	☐	Dual use research of concern
☒	☐	Plants

Methods

n/a	Involved in the study	
☒	☐	ChIP-seq
☐	☒	Flow cytometry
☒	☐	MRI-based neuroimaging

Antibodies

Antibodies used	Flow-cytometry antibodies:<br>1. EPCAM (anti-CD326 PE clone 9C4, Biolegend #324206, dilution 1:50), https://www.biolegend.com/en-gb/products/pe-anti-human-cd326-epcam-antibody-3757, stroma sorting.<br>2. CD45 (CD45 BV785, HI30 Clone (mouse), Biolegend #304048, dilution 1:50), https://www.biolegend.com/en-gb/products/brilliant-violet-785-anti-human-cd45-antibody-9325, stroma sorting.<br>3. DEC205 (APC anti-human CD205 (DEC-205), Biolegend #342207, dilution 1:50),https://www.biolegend.com/de-at/products/apc-anti-human-cd205-dec-205-antibody-5973, stroma sorting.<br>4. CD3 (FITC anti-human CD3, OKT3 clone (mouse), Biolegend #317306, dilution 1:50), https://www.biolegend.com/en-gb/products/fitc-anti-human-cd3-antibody-3644, stroma sorting.<br>5. CD3 (anti-CD3-PE clone SK7, Biolegend, 344805, 2ug/ml concentration), https://www.biolegend.com/en-gb/products/pe-anti-human-cd3-antibody-13257, T cell sorting.<br>CITEseq antibodies: The full list of TotalSeq-C antibodies used for CITE-seq is available in Supplementary Table 6 together with staining concentrations if available.<br>IBEX antibodies: All information relating to antibodies used for IBEX imaging, including dilutions, can be found in Supplementary Table 3.<br>RareCyte antibodies: Antibodies used for multiplex RareCyte staining were purchased from RareCyte: https://rarecyte.com/ and detailed in Supplementary Table 4 together with standard dilution (1:200).
Validation	Flow-cytometry antibodies: We have listed detailed antibody information as well as dilutions used in the field above. Please see references contained in the provided links to find available validations.

The IBEX antibody panel was put together after thorough testing of a large list of antibody candidates. All antibodies use in the study have been used in our prior publications or underwent validation on human tissue individually to ensure expected distributions and co-localisation where appropriate. Antibodies used for IBEX experiments have been deposited in the IBEX Imaging Community with reference imaging data: https://ibeximagingcommunity.github.io/ibex_imaging_knowledge_base/ [ibeximagingcommunity.github.io].
A thymus Organ Mapping Antibody Panel is available through the HuBMAP Organ Mapping Antibody Panel (OMAP) initiative. See OMAP-17 here: https://humanatlas.io/omap [humanatlas.io].
CITE-seq antibodies: TotalSeq-C Human Universal Cocktail has been validated by manufacturer (BioLegend, see https://www.biolegend.com/en-gb/products/totalseq-c-human-universal-cocktail-v1-0-19736). 13 additional TotalSeq-C antibodies were titrated according to the manufacturer's instructions and introduced into CITE-seq panel.
RareCyte antibodies: Antibodies used for multiplex RareCyte staining were purchased from RareCyte (immuno-oncology panel), see https://rarecyte.com/orionpanels/ and validated by the company.

Flow Cytometry

Plots

Confirm that:

☒ The axis labels state the marker and fluorochrome used (e.g. CD4-FITC).

☒ The axis scales are clearly visible. Include numbers along axes only for bottom left plot of group (a 'group' is an analysis of identical markers).

☒ All plots are contour plots with outliers or pseudocolor plots.

☒ A numerical value for number of cells or percentage (with statistics) is provided.

Methodology

Sample preparation

1. Stroma cell sorting: Briefly, tissue was finely minced and cell dissociation was performed using a mixture of liberaseTH (Roche, 05401135001) and DNAse-I (Roche, 4716728001) for ~30 mins in two rounds. Digested tissue was filtered through a 70 µm strainer and digestion was stopped with 2% FBS in RPMI media. Next, red blood cell lysis was performed on a cell pellet using the RBC lysis buffer from eBioscience™ (00-4333-57), after which samples were washed and counted.
To perform FACS sorting, cells were resuspended in the FACS buffer (0.5% FBS and 2mM EDTA in PBS), underwent blocking in TruStainFcX (422302, Biolegend) for 10 mins and were stained with a mixture of EPCAM (anti-CD326 PE clone 9C4, Biolegend #324206), CD45 (CD45 BV785, HI30 Clone (mouse), Biolegend #304048), DEC205 (APC a
2nti-human CD205 (DEC-205), Biolegend #342207) and CD3 (FITC anti-human CD3, OKT3 clone (mouse), Biolegend #317306) antibodies and DAPI for 30 mins. Upon staining, cells were washed and analysed using the Sony SH800 or Sony MA900 sorters with 130 µm nozzle.
2. T cell enrichment for CITEseq: Cells were thawed slowly by gradually adding 15 volumes of pre-warmed IMDM media and pelleted at 1700 rpm for 6 min at 4°C. After resuspending in PBS, cells were passed through a 70 µm strainer to remove clumps. Enrichment for viable cells was achieved using a magnetic bead-based dead cell removal kit (Miltenyi, 130-090-101). For this, cells were pelleted as before, washed with 1X Binding Buffer (part of kit, prepared with sterile distilled water) and resuspended in Dead Cell Removal MicroBeads (part of kit) at a concentration of 10^7 total cells/100ul beads. After incubation at RT for 15 min, cells were applied to an LS column (Miltenyi, 130-122-729), which was pre-rinsed with 3ml 1X Binding Buffer. The column was washed 4x with 3ml Binding Buffer and the flow through containing viable cells was collected. Cells in the flow through were pelleted and viability was confirmed using trypan blue. 2x10^6 viable cells were used for TotalSeq-C and anti-CD3-PE antibody staining. For this purpose, cells were washed with Cell Staining Buffer (Biolegend, 420201), pelleted at 600g for 10 min at 4°C, and resuspended in 90 ul Cell Staining Buffer. 10ul Human TruStain FcX Blocking solution (Biolegend, 422301) was added and cells were incubated for 10 min at 4°C. The TotalSeq-C Human Universal Cocktail 1.0 (Biolegend, 399905) was resuspended as described above, centrifuged at 14.000g for 10 min at 4°C and 25ul of the supernatant was added to the blocked cells. Individual TotalSeq-C antibodies were prepared as described above and 26ul of the master mix was added to each sample. To facilitate enrichment of immature and mature thymocytes via FACS, 10ul anti-CD3-PE (clone SK7, Biolegend, 344805) was added and samples were topped up with 40ul Cell Staining Buffer resulting in a total staining volume of 200ul. Samples were incubated for 30 min at 4°C in the dark. To wash off unbound antibody Cell Staining Buffer was added to the samples, and cells were pelleted for 10 min at 600g at 4°C. All supernatant was removed, cells were resuspended in Cell Staining Buffer, transferred to a new tube and pelleted as before. Cells were again resuspended in Cell Staining Buffer and pelleted and this wash step was repeated once more before cells were resuspended in 200ul MACS buffer (PBS + 2% FCS + 2mM EDTA) in preparation for sorting. 1ul PI was added for detection of dead cells and samples were sorted on a BD FACSAria III or BD FACSAria Fusion cell sorter using a 100 µm nozzle and a maximum flow rate of 4.

Instrument

Stroma sorting: Sony SH800 or Sony MA900.
T cell enrichment for CITEseq: BD FACSAria III or BD FACSAria Fusion cell sorter

Software

Stroma sorting: Data was analysed using FCSExpress7 version.
T cell enrichment for CITEseq: For the analysis software: CITE-seq was analysed using BD FACSDiva v8.0.1 and FlowJo v10.

Cell population abundance

Stroma sorting: CD45- cells constituted ~0.68-0.83% of the live liberase TH digested cells, while Thymic Epithelial cells composed ~0.19-0.25%.
T cell enrichment for CITEseq: The proportion of CD3- cells was 24.2-29.6% of live cells and the proportion of CD3+ cells was 70.4-75.9% of live cells depending on the donor.

Gating strategy

Stroma sorting: Small debris was removed using forward/side scatter, no multiplet filtering was performed to ensure large

Gating strategy

thymic epithelial cells (thymic nurse cells) can be included, dead cells were removed based on DAPI staining. CD45- cells were sorted to obtain stroma, while EPCAM+CD205- and EPCAM-CD205+ gates were pulled together to obtain total TEC fraction including cortical and medullary epithelial cells and sorted into solution of 2% FBS in PBS (Supplementary Fig.19A).
T cell enrichment for CITEseq: Cells were gated using forward/side scatter to remove doublets and debris, then dead cells were excluded based on PI staining. CD3- and CD3+ cells were collected separately in cooled IMDM + 50% FCS (Supplementary Fig.19B).

☒ Tick this box to confirm that a figure exemplifying the gating strategy is provided in the Supplementary Information.
