# Supplementary material for: A spatial human thymus cell atlas mapped to a continuous tissue axis
Source: Nature. 2024 Nov 20;635(8039):708–18. doi: 10.1038/s41586-024-07944-6 (PMC11578893; doi:10.1038/s41586-024-07944-6)
Supplement: Supplementary file 4 — Supplementary Tables 1–12. [file 41586_2024_7944_MOESM4_ESM.zip › 2023-09-16541B-s4/Supplementary Tables - legend.docx]

**Supplementary Table 1. Single cell metadata.** Composition of all dissociated datasets used in this study. "Age" denotes pcw for fetal samples and time after birth for paediatric samples. "age_numeric" was calculated as follows: for fetal samples: "age_numeric" = (pcw - 40) and for paediatric samples: "age_numeric" = (age in months).

**Supplementary Table 2. Spatial sample metadata.** Composition of all spatial data (Visium, IBEX, RareCyte, and RNAscope) used in this study, including

**Supplementary Table 3. IBEX antibody and imaging details.** Antibodies and imaging details for the custom IBEX panel implemented in this study.

**Supplementary Table 4. RareCyte antibody panel.** RareCyte antibody composition details.

[**Supplementary Table 5.**](https://docs.google.com/spreadsheets/d/1H7THP8xqCZlzOFRaxfEuTliFL82cDq47/edit?usp=sharing&ouid=117446946710264521619&rtpof=true&sd=true) **Results of two-way ANOVA.** Summary statistics from two-way ANOVA on cytokines with additional columns for cosine similarity. P-values were Bonferroni corrected.

**Supplementary Table 6. CITE-seq antibody panel.** Information about Biolegend TotalSeq-C antibodies used in this study.

**Supplementary Table 7. Cell counts by annotation level.** Absolute numbers of cells per annotation resolution (cell_type_level_1, 2, 3, 4, and 4_explore).

**Supplementary Table 8. CMA binning cutoffs.** CMA bin cutoffs to derive the binned CMA axis.

**Supplementary Table 9. Cell subsetting prior to cell2location mapping.** Details on cell types that were removed from fetal or paediatric single-cell datasets before deconvolution of spatial data.

**Supplementary Table 10. Cell Cycle Genes.** Genes excluded prior to scVI integration.

**Supplementary Table 11. IBEX CITE-seq KNN matching details.** Corresponding IBEX protein and scRNA-seq genes or CITE-seq proteins used for KNN matching.

**Supplementary Table 12. RNAscope details.** Probes, conditions, and controls used for RNAscope imaging of fetal thymus.
